# Fluorescent Imaging of Extracellular Fungal Enzymes Bound onto Plant Cell Walls

**DOI:** 10.3390/ijms23095216

**Published:** 2022-05-06

**Authors:** Neus Gacias-Amengual, Lena Wohlschlager, Florian Csarman, Roland Ludwig

**Affiliations:** Department of Food Science and Technology, Institute of Food Technology, University of Natural Resources and Life Sciences, Muthgasse 18, 1190 Vienna, Austria; neus.gacias@boku.ac.at (N.G.-A.); lena@wohlschlager.at (L.W.); roland.ludwig@boku.ac.at (R.L.)

**Keywords:** confocal laser scanning microscopy, enzyme localization, extracellular enzymes, fluorescence-labeling, *Phanerochaete chrysosporium*, poplar wood, secondary cell wall

## Abstract

Lignocelluloytic enzymes are industrially applied as biocatalysts for the deconstruction of recalcitrant plant biomass. To study their biocatalytic and physiological function, the assessment of their binding behavior and spatial distribution on lignocellulosic material is a crucial prerequisite. In this study, selected hydrolases and oxidoreductases from the white rot fungus *Phanerochaete chrysosporium* were localized on model substrates as well as poplar wood by confocal laser scanning microscopy. Two different detection approaches were investigated: direct tagging of the enzymes and tagging specific antibodies generated against the enzymes. Site-directed mutagenesis was employed to introduce a single surface-exposed cysteine residue for the maleimide site-specific conjugation. Specific polyclonal antibodies were produced against the enzymes and were labeled using N-hydroxysuccinimide (NHS) ester as a cross-linker. Both methods allowed the visualization of cell wall-bound enzymes but showed slightly different fluorescent yields. Using native poplar thin sections, we identified the innermost secondary cell wall layer as the preferential attack point for cellulose-degrading enzymes. Alkali pretreatment resulted in a partial delignification and promoted substrate accessibility and enzyme binding. The methods presented in this study are suitable for the visualization of enzymes during catalytic biomass degradation and can be further exploited for interaction studies of lignocellulolytic enzymes in biorefineries.

## 1. Introduction

The action of fungi and their secreted enzymes on various sources of plant biomass is of great economic interest. Firstly, their action degrades biomass such as wood and reed, which is crucial for the Earth’s carbon cycle, and secondly for their use in biofuel production and biorefineries. However, studying their catalytic activities, in particular the interaction of several enzymes on the solid structure of plant cell walls, is impeded by the heterogeneity of the substrate and its structural complexity. Simple enzymatic assays are not suitable to study the complex and multifaceted interactions of dozens of different enzymes on plant cell walls. In order to investigate their localization and interactions with plant cell wall biopolymers, an adequate visualization on the nano- and meso-scale is necessary [1].

Fluorescence microscopy methods have become one of the major contributors to the study of in situ enzyme localization and interactions within the substrate. These imaging techniques offer several advantages as they are non-invasive, highly sensitive, and offer high versatility, as multiple fluorescence probes can be monitored simultaneously [2]. In addition to directly labeling enzymes, which may alter the properties of native enzymes, conjugated antibodies are a powerful alternative to study the extracellular secretion of fungal lignocellulolytic enzymes during the stages of biomass decay [3]. Nevertheless, these techniques require protein conjugation with a fluorophore via specific chemical groups. Maleimide conjugation relies on the click-reaction between a thiol and a maleimide group generating a stable thioether bond. This conjugation approach is one of the most popular chemistries for the site-selective modification of cysteine residues for bioconjugation purposes, due to cysteine’s relatively low abundance in folded proteins (1–2%) [4] and the enhanced nucleophilicity of the sulfhydryl group [5]. Another conjugation method relies on the N-hydroxysuccinimide (NHS) ester activating group. This approach is one of the oldest and most versatile techniques for protein conjugation. During the reaction, the ester-containing reagents react with nucleophiles, causing the release of the NHS group to form an acylated product. In proteins, the NHS crosslinking reagents couple generally with the α-amines at the N-terminals and the ε-amines of lysine side chains [6]. This conjugation approach is the most popular for labeling large proteins such as antibodies (150,000 g mol^−1^) because usually, more than 80 lysine amines are available for the reaction [5]. Despite its simplicity and ease of use, NHS ester crosslinkers are unspecific and cause heterogeneous labeling [7]. Among the fluorescence imaging techniques, confocal laser scanning microscopy (CLSM) has been commonly used to visualize native lignocellulose structures [8], the effect of pretreatment methods on cell wall structures [9,10], and the adsorption of various enzymes to simple [11,12,13] and complex lignocellulosic substrates [14].

The enzymatic hydrolysis of lignocellulose substrates is a heterogeneous process involving 4 main steps: (1) a fast adsorption of the enzyme onto the substrate; (2) the alignment of the active site of the enzyme with the substrate; (3) enzymatic hydrolysis; and (4) enzyme desorption and dissociation from the substrate. Several substrate and enzyme-related properties have been identified to affect the adsorption and the hydrolysis process. Substrate factors comprise both physical features such as cellulose crystallinity, degree of polymerization, porosity, and availability of accessible surface area, and chemical features such as hemicellulose and lignin presence in complex lignocellulosic materials. To investigate enzyme adsorption, simplified cellulose models (Avicel PH-101, cotton linters, α-cellulose), as well as isolated lignin using various extraction strategies and lignocellulose origins, have been widely used [15,16]. However, during the isolation process, lignin and cellulose structures are modified to some extent and the reported enzyme–substrate interactions in the literature might not be representative of the native binding. Complex lignocellulosic materials contain cellulose (33–51%), hemicelluloses (19–34%), pectin (2–20%), and lignin (20–40%) arranged in a honeycomb-like structure formed by chemically heterogeneous layers sequentially deposited during cell growth and differentiation [17,18]. The primary cell wall (P) is the first layer to be formed [19]. It is highly elastic and is composed mainly of randomly distributed cellulose fibers in a matrix with hemicelluloses and pectin. The middle lamella (ML) cements two adjacent cells. Initially, it is mainly composed of pectin, but in later growth stages, both middle lamella and the primary cell wall become lignified, forming the compound middle lamella (CML). Once the cell reaches its final size, the secondary cell wall layer (SCW) is deposited. SCW is subdivided into three sub-layers (S1, S2, S3) differing in thickness and cellulose microfibril orientation. S1 is the first layer to be deposited in the vicinity of the primary cell wall (P), forming the thinnest of the S layers [17,19]. The S2 layer containing most of the lignin is the thickest of the secondary cell wall layers and has the greatest influence on the mechanical properties of the cell wall. The last layer to be deposited in the vicinity of the cell lumen is the S3 layer. This layer is relatively thin (0.1 µm) and possesses the lowest lignin content. Generally, lignification is initiated with the formation of the secondary cell wall [20]. However, lignin composition differs between species and plant tissues. Lignin is formed from aromatic building blocks (mainly p-hydroxyphenyl (H), guaiacyl (G), and syringyl (S) units) which impregnate plant cell walls, providing a unique resistance toward chemical and enzymatic degradation. Lignin acts as a steric hindrance, physically blocking enzyme access to cellulose microfibrils and additionally causing unspecific enzyme adsorption [21,22]. Thus, to promote lignocellulose conversion into fermentable sugars, a pretreatment step is often required to remove, alter, or reduce lignin content. Several pretreatment strategies, such as physical, physicochemical, chemical, biological, or a combination of these, have been investigated. Among them, alkali reagents such as sodium or potassium hydroxide have been widely applied to delignify lignocellulosic materials as they cleave α-ether linkages forming between lignin and hemicelluloses, and de-esterify intermolecular ester bonds [23,24].

Fungi have evolved an extensive portfolio of enzymes able to open the complex plant cell wall architecture, decomposing the different polymers into sugars. One of the best studied fungi is the white rot *Phanerochaete chrysosporium*. When grown on ball-milled aspen wood as a carbon source, this fungus secretes 79 different extracellular glycosyl hydrolases (GHs) and auxiliary enzymes [25]. Cellobiohydrolases belonging to the glycoside hydrolase family 6 (CBHII, Cel6A) degrade cellulose microfibrils in a processive manner from the non-reducing ends, releasing cellobiose units [24]. CBHII consists of a catalytic domain (CD) and an N-terminal binding carbohydrate-binding module (CBM) type 1 with a high affinity toward crystalline cellulose [26]. Family 45 glycoside hydrolases (GH45) are endoglucanases small in size (~20–45 KDa) and active on a wide range of substrates including both β-1,4 or β-1,3/1,4-glucans [27,28]. Although some GH45 members contain carbohydrate-binding modules (CBM), fungal Cel45 from *P. chrysosporium* consists only of a catalytic domain. In addition to GHs acting on polysaccharides, a variety of redox enzymes are involved in lignocellulose depolymerization, acting directly on the polymers or by producing secondary metabolites and enzymatic co-substrates. Lytic polysaccharide monooxygenases (LPMOs) attack the crystalline regions of cellulose microfibrils, providing new accessing points for GHs. LPMOs depend on the reduction by exogenous electron donors such as cellobiose dehydrogenase (CDH), or phenolic mediators, and require H_2_O_2_ as a co-substrate to be active [29,30]. In the secretome of *P. chrysosporium*, the copper-containing metalloenzyme glyoxal oxidase (GLOX) has been identified to produce H_2_O_2_ that is also utilized as a co-substrate for lignin-degrading peroxidases [31]. Although the extracellular sets of the lignocellulolytic enzyme from fungi like the model organism *P. chrysosporium* have been investigated for decades, studies on the local distribution of these enzymes on their native substrates are rare.

This study investigates two different approaches to fluorescently detect the binding of a set of fungal hydrolases and oxidoreductases from the white rot *P. chrysosporium*. The first approach is the direct tagging of the heterologously produced enzymes using maleimide-activated fluorescent dyes. The second approach is the indirect detection of those enzymes by specific polyclonal antibodies conjugated by NHS ester-activated fluorescent dyes. We compare the efficiency of the fluorescence conjugation between both approaches and the enzyme binding pattern on α-cellulose, poplar wood cell wall, and partially delignified poplar cell wall treated with alkali reagents.

## 2. Results

### 2.1. Enzyme Conjugation with Fluorescent Dyes

A set of two hydrolases (CBHII and Cel45A) and three oxidoreductases (LPMO, CDH, and GLOX) from the white-rot *P. chrysosporium* were heterologously produced to be tagged to DyLight dyes via maleimide coupling. This approach specifically targets accessible sulfhydryl groups in proteins, which are not available in the selected native enzymes. Therefore, a surface-exposed cysteine was introduced by site-directed mutagenesis into each enzyme to allow specific coupling of the fluorophore. This was done by exchanging a surface-exposed residue with a similar physicochemical property (Ser, Ala), or by introducing a cysteine residue at the C-terminus (Cel45A) (Table 1, Supplementary Materials S1 and S2). The coupling reactions typically had a high recovery of fluorescent-tagged enzymes with yields ranging from 71–96%. To test whether the catalytic activity was affected by maleimide coupling, the enzymatic activity of conjugated and non-conjugated CBHII, CDH, and GLOX were compared. For all enzymes investigated, no significant difference between the labeled and non-labeled enzyme was found, which indicates no interference of the conjugate with the enzyme’s catalytic activity nor a detrimental effect on protein stability (Supplementary Method S2). Labeling efficiencies were evaluated by measuring the UV/Vis absorption spectra of the conjugated enzymes. The reaction of maleimide towards free thiols yielded a sufficient degree of conjugation with the DyLight fluorescent dye for subsequent CLSM experiments ranging from 0.14 moles of dye per moles of protein for LPMO9D to 1.04 moles of dye per moles of protein for CBHII.

Several factors could contribute to the varying labeling efficiencies observed for the different enzymes investigated. Although the presence of cysteine residues in proteins is rather low (1–2%) [4], native cysteines could interfere with the crosslinking, affecting the overall labeling. While no free cysteine residues are present in the protein structures of CBHII, or Cel45A, the structures of LPMO9D, CDH, and GLOX harbor native free cysteine residues that could potentially react with the mutated residue and thereby block its availability for the maleimide coupling reaction. For instance, the protein model of GLOX, generated using the crystal structure of the related galactose oxidase (GAOX) from *Fusarium* sp. as a template (pdb: 1GOF), showed nine cysteine residues that could be susceptible to reduction by TCEP, affecting the labeling efficiency. Deviations could also originate from varying labeling conditions, such as a slight change in pH, temperature, or the presence of TCEP that can inhibit the reactivity of thiol-reactive dyes, reducing the labeling yield.

### 2.2. Antibody Conjugation with Fluorescent Dyes

**Figure 1:**
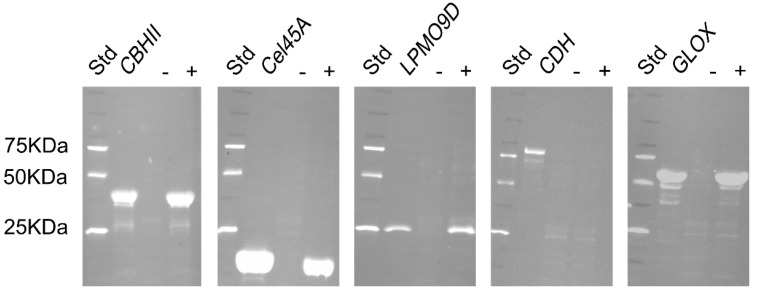
Western blot analysis of conjugated rabbit polyclonal antibodies with DyLight dyes against *P. chrysosporium* hydrolases and oxidoreductases. Std lane corresponds to the Precision Plus Protein WesternC standard ladder (Bio-Rad, Hercules, CA, USA). *P. chrysosporium* culture supernatant after ~7 days of fermentation showing no detectable activity of the investigated enzymes was used as a negative control (−). The culture supernatant was supplemented with the targeted enzyme and was used as a positive control (+). Conjugated antibodies (1:5000) were incubated in PBS supplemented with 1% BSA for 2 h at 21 °C and washed for 5 min each in PBS containing 0.1% (*v*/*v*) Tween 20 3 times. Antibody binding was detected by exciting the membrane with Cy 2 and Qdot 625 excitation/ emission settings corresponding to DyLight D550 (CBHII; CDH; GLOX) and DyLight D633 (Cel45A, LPMO9D) respectively.

Polyclonal antibodies against hydrolases and oxidoreductases from *P. chrysosporium* were raised in New Zealand white rabbits at ThermoFisher Scientific facilities using purified deglycosylated enzymes for the immunization process. The antibodies were purified by affinity chromatography using the target enzymes and antibody titers evaluated by indirect ELISA at ThermoFisher Scientific facilities. ELISA titers (Table 2) indicate the minimum concentration at which the purified antibodies can effectively detect the antigens, thus lower concentrations indicate higher sensitivities. The polyclonal antibody against Cel45A showed the highest sensitivity (39 ng mL^−1^) and AbCBHII the lowest (625 ng mL^−1^). To validate antibody specificity and selectivity, a Western blot was performed. A supernatant obtained from a *P. chrysosporium* liquid culture, which did not contain detectable activities of the investigated enzymes, was used as a negative control. The same supernatant supplemented with the targeted enzymes was used as a positive control to evaluate antibody specificity against the antigen in this matrix (Figure 1). All antibodies were shown to be highly specific as they were able to recognize and bind to their target antigens and positive controls using a 1:5000 dilution. The selectivity detected by Western blot was different for the studied antibodies. In agreement with the low indirect ELISA titers, AbCel45A and AbLPMO9D showed no bands in the negative control, which suggests a high selectivity. The AbCBHII with the highest ELISA titer also showed no bands in the negative control, which indicates a high selectivity but unfortunately a low sensitivity. In the case of AbCDH and AbGLOX, both showed some additional weaker bands, indicating a lower selectivity towards the targeted antigen. To covalently tether the polyclonal antibodies, NHS ester chemistry was chosen as it is a routinely used procedure for fluorescently labeling antibodies. The yield of recovered antibodies was in the range of 56–78%. The success of NHS conjugation was evaluated by measuring the UV/Vis absorbance spectra of the conjugated antibodies (Figure S4). Distinct labeling efficiencies were obtained ranging from 0.94 to 4.91 moles of dye per mole of protein (Table 2). NHS conjugation chemistry is based on the reaction between the NHS ester moiety of the fluorophore with α-amines at N-terminals and ε-amines of lysine side chains present in the native protein. In the case of IgGs, more than 80 lysine residues are present in the structure [32], which could be potentially be targeted by the NHS ester moiety and explain the multiple fluorophores per enzyme molecule.

### 2.3. Enzyme Adsorption Studies on Defined Substrates

Different cellulose preparations, including Avicel or α-cellulose, as well as lignin isolated by different extraction procedures, are commonly used as model substrates to study the adsorption of enzymes on lignocellulosic biomass [33,34,35]. In this study, commercial α cellulose generated by alkali treatment of cotton linters was selected to investigate enzyme localization and distribution by confocal laser scanning microscopy (CLSM) using two different approaches: (i) directly labeled enzymes; or (ii) applying native enzymes and specific conjugated antibodies. For both methods, equal incubation times and reaction conditions were applied to allow a direct comparison of both methods. The preincubation was set to 20 min, as previous reports demonstrated that enzyme adsorption tends to be rapid, with a half maximum of adsorbed enzyme binding within 1–2 min. Imaging was performed within a maximum 30 min to prevent excessive degradation by the enzymes [36]. Representative image sections of α-cellulose fibers considering the size, shape, and position relative to the coverslip were selected for further analysis and comparison of the different labeling strategies. The results are shown as a sum projection of z-stacks with a z-step size of 1 µm. The individual pictures are displayed as a montage in Figures S7 and S8. Additionally, no significant levels of dye photobleaching could be observed during the imaging protocol.

#### 2.3.1. Detection of Directly Tagged Enzymes on α-Cellulose

**Figure 2:**
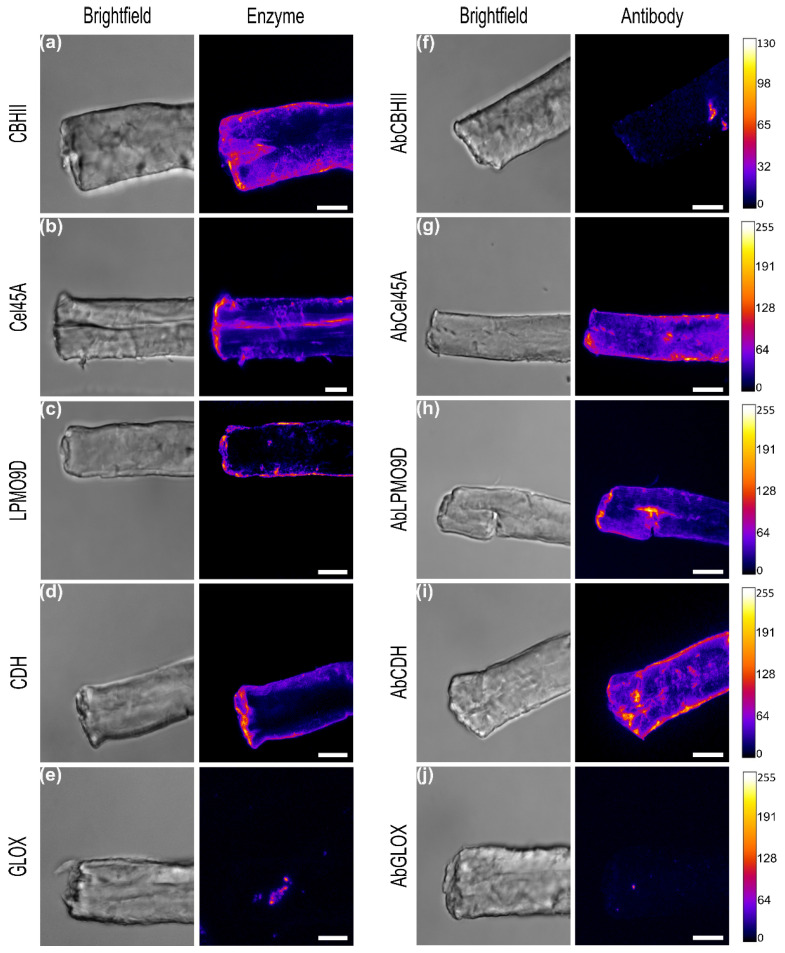
Binding of directly labeled enzymes (left, (**a**) CBHII, (**b**) Cel45A, (**c**) LPMO9D, (**d**) CDH, (**e**) GLOX) and indirect detection by conjugated polyclonal antibodies (right, (**f**) AbCBHII, (**g**) AbCel45A, (**h**) AbLPMO9D, (**i**) AbCDH, (**j**) AbGLOX) on α-cellulose fibers. Images are sum projections of acquired z-stacks. Brightfield images show α-cellulose structure. Fluorescence images are intensity-coded using Fire LUT. Images are displayed with the same fluorescence intensity scale (0–255) except for AbCBHII (0–130). Scale bars correspond to 10 µm.

In general, the highest amount of bound enzyme, and therefore the highest fluorescence, was detected at the outermost layer of the fibers, especially on the edges rather than within the fibers. A high surface loading using the directly labeled enzymes could be observed for CBHII and Cel45A, while a lower signal was found for LPMO9D and CDH (Figure 2a–d). Almost no signal of the bound enzyme could be observed for GLOX (Figure 2e).

For CBHII, the highest fluorescence was detected at the outermost fiber layers, including broken structures on the fiber edges but also on fiber surface. This enzyme contains an N-terminal carbohydrate-binding module type 1 (CBM1) with a high affinity towards the crystalline regions of cellulose. The binding of the isolated CBM1 of Cel6A from *Trichoderma reesei* has been investigated by CLSM and high-resolution techniques like photoactivated localization microscopy (PALM), showing that it preferentially binds to the crystalline region of the linter, although it could also bind to less ordered regions with a lower affinity [37,38].

In contrast to CBHII, the highest signal of labeled Cel45A was observed on the fiber edges, probably a region with a high roughness facilitating enzyme accessibility with a preference for amorphous regions (Figure 2b). In addition to the edges, Cel45A was detected on small protruding structures and along cavities running parallel to the outermost layer. The small size of the enzyme (18 kDa) has been suggested to facilitate its diffusion into the fiber structure, as well as into the pores of the lignocellulose matrix [39]. Due to the high labeling efficiency (0.86 moles of dye/mole protein, Table 1), the laser intensity and the gain of the detector were reduced to avoid image saturation (Table S2). Hidayat et al. showed that Cel45A endoglucanase from *Humicola insolens* had a stronger binding preference to dislocations present in the fiber rather than in the surroundings of the cell wall [12]. Other studies showed that fiber dislocations are mechanically weak, with different cellulose microfibril angles compared with the surrounding cell wall. Thus, those regions could act as the starting point for fiber fracture by hydrolases [40,41].

In the case of LPMO9D (Figure 2c), most of the emission was detected at fiber side walls rather than on top surfaces. Fungal LPMO belonging to family AA9 have a flat substrate-binding surface with a catalytic site optimized for attacking crystalline regions of cellulose, causing local disruption in these ordered regions [41]. Previous studies investigating the binding of LPMO9C from *Neurospora crassa* by atomic force microscopy indicated that the binding and dissociation of the enzyme was 4.5-fold higher on the side faces of the cellulose nanocrystals than on the crystal’s top face [42]. However, while *Nc*LPMO9C possesses a CBM, LPMO9D lacks this domain, and the substrate specificity and preference for certain crystalline regions might also be different for this enzyme. 

Similar to the results obtained with CBHII, CDH (Figure 2d) showed the highest signal on the outermost part of the fibers, including cavities and small cracks protruding from the edge, and only minor emissions could be detected at the fiber’s top surface. No enzyme binding to α-cellulose could be observed for GLOX (Figure 2e), suggesting that this enzyme is not selectively binding to cellulose surfaces. Therefore, GLOX was used as a negative control for subsequent binding experiments.

#### 2.3.2. Indirect Detection of Enzymes by Labeled Antibodies

Compared to the results of the directly labeled enzymes, the signal of the indirect detection using conjugated antibodies was weaker and a higher signal at the fiber’s top surface was detected, indicating minor unspecific binding of the antibodies (Figure S5). In particular, the signal of CBHII using the conjugated antibody was weak (Figure 2f). Several factors could contribute to the weak signal. First, the labeling yield was low, with only 1.86 moles of dye per mole of antibody. Secondly, the antibody-antigen complex formation might inhibit the efficient binding of CBHII on α-cellulose and its posterior substrate recognition by blocking surface moieties of CBHII that are crucial for its binding affinity. To identify possible antigen regions involved in antibody recognition, three in-silico B-cell epitope prediction tools were used: BepiPred-2.0 [43] based on the antigen sequence, DiscoTope-2.0 [44] based on the antigen 3D conformation, and EpiPred [45], which aligns both the antigen and the selected antibody structure (Supplementary Method S3). Using the DiscoTope-2.0 algorithm only, epitopes located on the catalytic domain could be determined due to the absence of the linker and the CBM in the crystal structure of CBHII (pdb: 5XCY). BepiPred-2.0 predicted epitopes on the CBHII sequence located on the CBM domain and the linker region as well as in the catalytic domain, which might interfere with binding to the substrate.

In the case of AbCel45 (Figure 2g), the highest intensity was observed at the outermost part of α-cellulose. However, a minor fluorescence was also detected on the surface of the fiber and in small channels formed at the top area. Compared to the results obtained using directly labeled Cel45A, we observed a similar binding pattern. Both methods highlight the preferential binding of this enzyme to fractures and protruding fractions on the fiber’s surface. AbLPMO9D (Figure 2h) emitted the highest fluorescence in a fissure within the fiber’s top surface as well as on fiber edges. As observed for the directly labeled CDH, AbCDH detected the antigen at the outermost region of the fiber, including the edges, which show some kinks and open structures together with surface protruding fractions (Figure 2i). In a previous study, Igarashi et al. investigated the secretion of CDH of the fungus *P. chrysosporium* on Whatman CC-41 cotton fibers using a polyclonal antibody raised against CDH and an anti-mouse IgG labeled with fluorescein isothiocyanate. The results showed that most of the adsorbed enzymes localized on fiber cracks and only minor amounts of the enzyme adsorbed to the smooth surface [46]. 

As observed for the directly labeled GLOX, only minor unspecific binding of the polyclonal antibody could be detected, and no significant binding of GLOX to the cellulosic substrate (Figure 2j).

### 2.4. Enzyme Adsorption on Native Poplar Cell Walls

In our effort to study enzyme adsorption to native lignocellulose samples we used juvenile poplar wood (5 years) which is characterized by a lower fiber cell wall crystallinity, a larger microfibril angle, and thinner secondary cell walls [8]. To study enzyme adsorption, transverse cross-sections of fiber cells consisting of alternating lumen openings surrounded by rougher cell wall surfaces were selected. It is worth noting that protein adsorption on solid substrates is a dynamic process that involves continuous structural changes in proteins as they reversibly adsorb and desorb. However, as the process progresses, numerous rearrangements between the two states may compromise the protein’s structural integrity, resulting in irreversible structural alterations and protein activity loss [47,48]. For most of the enzymes, the highest fluorescence intensity was localized on the inner part of the cell wall, corresponding to secondary cell wall layers (SCW) S3 and S2 sub-layers. Nonetheless, unspecific binding to lignin-rich regions was detected to different extents for the enzymes investigated, which is in good agreement with the results of Ding et al. [49] Besides substrate-related factors, enzyme surface properties and hydrophobicity strongly influence their affinity towards lignin. Therefore, physicochemical properties of the enzymes used within this study that are potentially influencing their binding behavior include protein size, aliphatic index, and solvent accessible hydrophobic surface area as well as their experimentally determined isoelectric points, all of which were evaluated (Table 3).

#### 2.4.1. Detection of Directly Tagged Enzymes on Cell Walls

**Figure 3:**
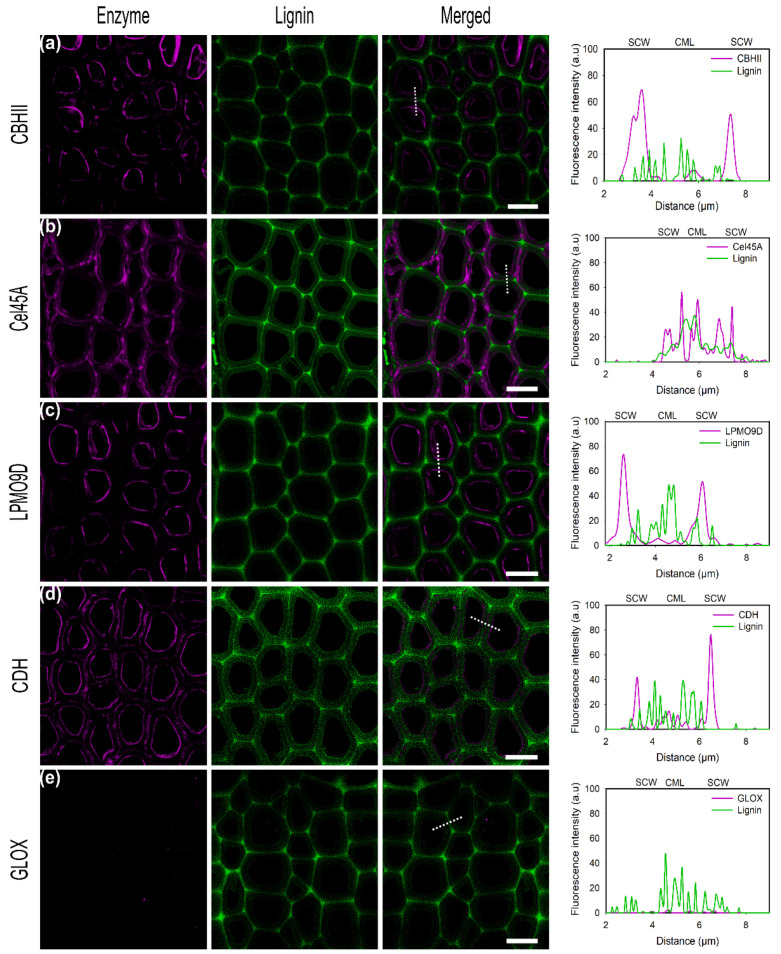
CLSM images of conjugated enzymes (**a**) CBHII, (**b**) Cel45A, (**c**) LPMO9D, (**d**) CDH, (**e**) GLOX on poplar cross-sections showing fiber cells. Enzyme fluorescence is colored in magenta. Lignin autofluorescence is shown in green. Merged column displays the overlay of enzyme and autofluorescence channels. Graphs showing the overlay profile (dash line) of line scans over fiber cell wall, showing secondary cell walls (SCW) and compound middle lamella (CML). The brightness was enhanced to allow proper visualization using the same factor. Scale bars correspond to 10 µm.

CBHII binds at the inner parts of the cell wall but additionally unspecifically binds to lignin-rich regions, as cell corners and middle lamella could be observed (Figure 3a). In previous studies, the CBM of CBHII was shown to increase the adsorption on lignin-rich areas [53]. A binding study of Cel45A from *Melanocarpus albomyces* Rahikanien et al. showed that the presence of CBM increases enzyme adsorption to lignin, especially at a high pH, and suggested hydrophobic interactions to be the major driving force governing unspecific binding [22]. Studies have identified the highest hydrophobic patch on the CBM of *T. reesei* Cel7A, highlighting the pivotal role of hydrophobic interactions [54]. Investigations by molecular dynamic simulations by Vermaas et al. showed that lignin could outcompete cellulose during CBM binding recognition via hydrophobic interactions interfering with cellulose-CBM 1 contact by simulations [55]. In this study, the analysis of the physicochemical properties of the enzymes used showed that CBHII has the highest predicted hydrophobic surface area (4058 Å^2^), which could explain the high degree of unspecific binding to lignin by hydrophobic interactions (Table 3). 

For the endoglucanase Cel45A (Figure 3b), a high fluorescence signal, over almost the entire cross-section of the cell wall, was observed with the maximum on the inner cell wall but also in lignin-rich areas such as cell corners and middle lamella. The small size of the GH45 enzyme has been hypothesized to facilitate its penetration into small pores and cavities within the lignocellulose matrix, allowing it to gain better access towards its substrate [38]. Native *Ma*Cel45A showed a slightly higher binding towards wheat straw enriched lignin than for microcrystalline cellulose (Kd = 16.39 µM, Kd = 18.86 µM respectively). *P. chrysosporium* Cel45A lacks a CBM domain, suggested to govern enzyme unspecific binding to lignin by hydrophobic interactions. Instead, electrostatic repulsion forces have been identified to contribute to enzyme interactions with the substrate [22]. 

In regard to LPMO9D, as well as for CBHII and CDH, the maximum fluorescence signal was detected on the inner cell wall which correlates to the position of lower lignin autofluorescence and thereby demonstrates the affinity of this enzyme towards cellulosic substrates (Figure 3c). In this study, LPMO9D was applied in its oxidized state in the absence of a suitable reductant. However, previous studies showed that LPMO substrate affinity is significantly increased by the reduction of the active site copper. Kracher et al. showed that LPMO binding capacity towards dispersed PASC was approximately two-fold higher as well as eight times faster in the presence of ascorbic acid as the reductant (Kd = 4.4 ± 1.0 and 9.5 ± 2.2 µM, respectively) [56]. Although in this study no reductant was added to LPMO9D, the incubation time used (20 min) was sufficient to almost reach the binding equilibrium. 

CDH showed the highest intensity at the inner part of the cell wall corresponding to the S3 layer as also observed for CBHII (Figure 3d). Although some fluorescence could be detected in the compound middle lamella regions (CML), the intensity found within the cell walls was three times higher (SCW), suggesting only minor unspecific binding towards lignin. Although CDH from *P. chrysosporium* does not contain a CBM as found with many CDHs from ascomycetes, a strong affinity towards cellulose has been demonstrated in previous studies [57]. The observed binding pattern, with only a low degree of unspecific binding, agrees with enzyme physicochemical properties as CDH showed the lowest aliphatic index (Table 3).

In contrast to the other enzymes investigated in this study, no binding of GLOX on poplar sections could be observed as also found using α-cellulose as a substrate (Figure 3e). Studying GLOX from the closely related species *Phanerochaete crassa*, Takano et al. found that GLOX remains associated with the fungal cell wall localized mainly at the hyphal tips and is not released into the culture filtrate [58]. As we could not detect any significant binding of GLOX to α-cellulose nor to native biomass, this could indicate that GLOX is adapted to specifically associate and bind to the macromolecules of the fungal cell wall or that GLOX is freely diffusing.

##### Cellulose Binding Specificity

To promote enzyme accessibility to cellulose, a pretreatment step involving lignin and hemicellulose structural modification and partial removal, reduction of cellulose crystallinity, and an increase in material porosity is required. Therefore, wood samples were treated with 10% KOH for 4 h at 80 °C, which led to a swelling of the cell wall as reported in the literature [59,60,61]. In addition, the lignin intensity detected by the autofluorescence emission between 405–550 nm increased for the pretreated samples, suggesting a higher exposure of lignin aromatic residues [62].

**Figure 4:**
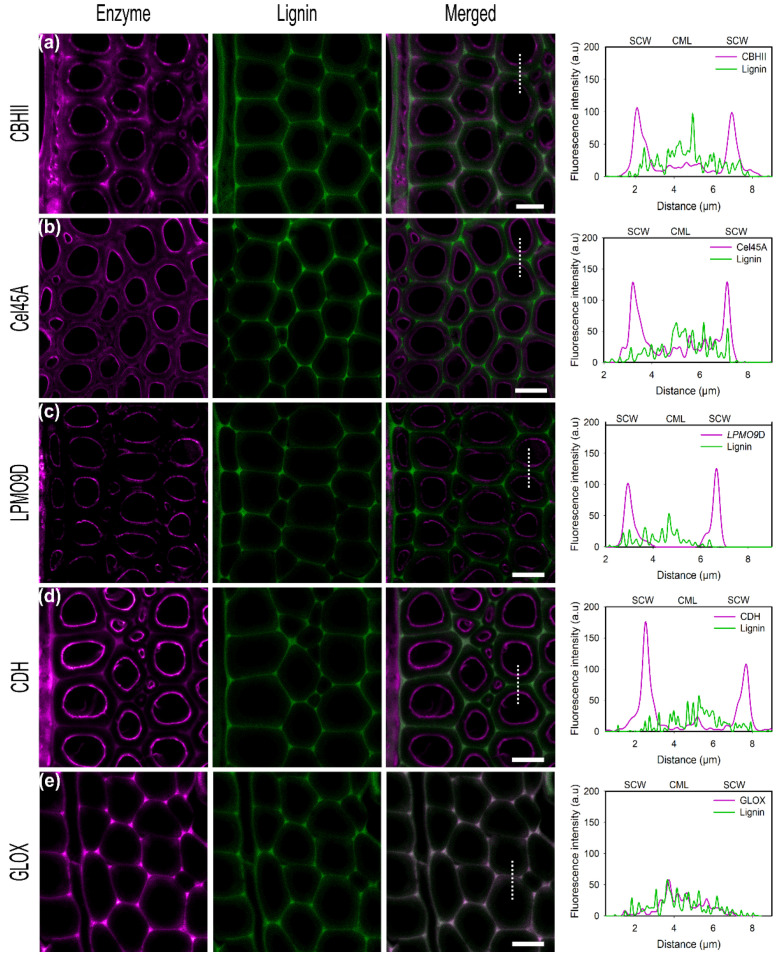
CLSM images of conjugated enzymes (**a**) CBHII, (**b**) Cel45A, (**c**) LPMO9D, (**d**) CDH, (**e**) GLOX on delignified poplar cross-sections showing fiber cells. Enzyme fluorescence is colored in magenta. Lignin autofluorescence is shown in green. The merged column displays the overlay of enzyme and autofluorescence channels. Graphs show the overlay profile (dash line) of line scans over the fiber cell wall, showing secondary cell walls (SCW) and compound middle lamella (CML). Scale bars correspond to 10 µm.

The highest fluorescence upon incubation of CBHII with pretreated poplar was observed on the inner parts of the cell wall (Figure 4a). However, a higher degree of potentially unspecific binding was also detected in cell corners and in middle lamella, which was higher than levels previously observed for untreated poplar. As described above, CBMs play a pivotal role in the enzyme affinity to lignin. As hemicelluloses are more sensitive to thermal and chemical treatment, high polymer portions are removed during pretreatment with alkali reagents, and lignin may be more exposed, increasing the unspecific binding. In a comparison between fungal cellulases, cellulosomes, and CBMs, Ding et al., found that acid delignification increased the overall enzyme binding to secondary walls in general but to a lower extent for enzymes featuring a CBM domain [49]. 

In the case of Cel45A, even if the highest fluorescence intensity is detected at the inner parts of the cell wall after pretreatment, unspecific binding also increased in comparison to untreated poplar (Figure 4b). Furthermore, the fluorescence pattern differs after treatment. While for untreated poplar, unspecific binding seemed localized in ML regions and not on the cell wall surface, after pretreatment higher unspecific binding covering the whole fiber cell wall was detected. Those differences associated with alkali treatment suggested that after incubation, structural features and pore openings were modified, potentiating enzyme penetration into S3 and S2 layers.

As for non-delignified samples, LPMO9D preferentially binds to SCW regions after pretreatment (Figure 4c). It has been suggested that radicals generated during pretreatment could be used as possible electron donors for the reduction of LPMO9D, boosting its activity [63,64]. However, in our case, only a minor increase in fluorescence intensity in SCW regions compared to untreated poplar was observed.

For the native poplar wood, we observed that most of the labeled CDH (Figure 4d) binds to the inner part of the cell wall, corresponding to the S3 layer and in minor proportion, some unspecific binding within CML (around 20–40% less fluorescence intensity) was detected. In regards to pretreated slices, conjugated CDH followed the same binding pattern. However, the detected intensity was two-fold higher than for native samples when using the same excitation and emission settings (Table S3). Moreover, some fluorescence is also observed in cell wall filaments detached during microtome sectioning. Interestingly, the unspecific binding to the middle lamella increased in comparison to the untreated poplar slice. Inner SCW showed a three-fold higher signal than CML and a 2-fold higher signal than cell corners. Nevertheless, this could be explained by the higher surface area exposed on cell corners rather than in the middle lamella. In addition, cell corners are rich in lignin G-units, which have been shown to promote unspecific binding via hydrophobic bonding [65].

In contrast to the experiments with the untreated samples, a fluorescence signal with GLOX was observed. However, the overlay of this signal with the autofluorescence of lignin shows clearly the spatial colocalization of GLOX and lignin. Therefore, the binding of GLOX to the pretreated wood sample is potentially caused solely by unspecific interactions with the exposed lignin residues (Figure 4e). 

##### Suppression of Unspecific Binding

**Figure 5:**
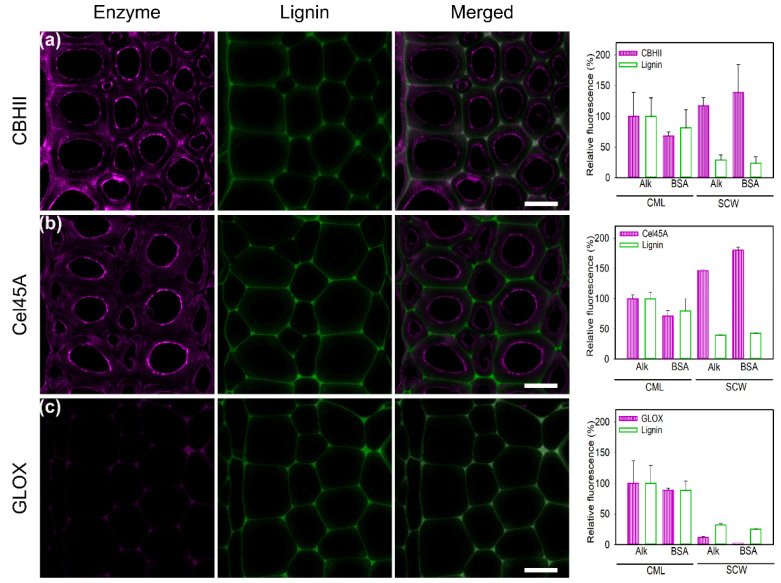
CLSM images of conjugated (**a**) CBHII, (**b**) Cel45A, and (**c**) GLOX on delignified poplar samples after incubation with 1% BSA. Enzyme fluorescence is colored in magenta. Lignin autofluorescence is shown in green. The merged column displays the overlay of enzyme and autofluorescence channels. Histograms show the maximum relative fluorescence intensity, expressed in percentage, of the alkali-treated compound middle lamella (CML), designated as 100%, and its comparison after 1% BSA preincubation. Scale bars correspond to 10 µm.

Various additives, such as non-ionic surfactants (Tween 20, Tween 80) and non-catalytic proteins (BSA), have been reported as “shielding agents”, which reduce enzyme unproductive binding to lignin [66,67]. In this study, the effect of BSA preincubation with CBHII, Cel45A, and GLOX on alkali pretreated poplar slices was tested (Figure 5). The effect of BSA incubation on enzyme binding and lignin autofluorescence is expressed as a percentage of the fluorescence signal, with the intensity of the CML region in alkali-treated poplar designated as 100%. In the case of CBHII binding (Figure 5a), BSA treatment resulted in a 30% decrease in unspecific binding in lignin-rich areas (CML) and a 60% increase in SCW binding. The same pattern was found for lignin autofluorescence, with a reduction of 20% in CML. We hypothesize that the addition of BSA quenches lignin autofluorescence. In the case of Cel45A (Figure 5b), BSA incubation resulted in significant changes in the binding pattern in SCW regions (Figure 5b). While there was a slight decrease (30%) in CML regions, there was an increase of 80% in SCW enzyme binding. The observed changes may be attributed to the enzyme’s small size, which may facilitate enzyme penetration after alkali pretreatment, enhancing the enzyme’s binding to cellulose and hemicelluloses, where the enzyme is also active. For lignin autofluorescence, a 30% decrease was observed upon addition of BSA to CML and minor changes were distinguished in SCW. Unspecific binding to CML regions by GLOX was reduced 20% after BSA treatment, as was lignin autofluorescence (Figure 5c). In SCW regions, the signal was reduced by up to 90%, indicating a major reduction in unspecific binding. Overall, BSA reduced the unspecific binding to CML regions, while slightly increasing the specific binding to SCW regions as previously reported [61,62]. Although the difference in adsorption between cellulases and BSA is not fully understood, it is believed that BSA’s hydrophobic sites bind to fatty acids as well as the hydrophobic surface of lignin, acting as “lignin blockers” and increasing hydrolytic activities [68]. The BSA structure displays 7 hydrophobic patches of different sizes (50–450 Å^2^) exhibiting higher binding capacities to lignin surfaces than other proteins and fast adsorption rates of BSA have been reported [54].

#### 2.4.2. Indirect Detection of Enzymes on Plant Cell Walls by Conjugated Antibodies

**Figure 6:**
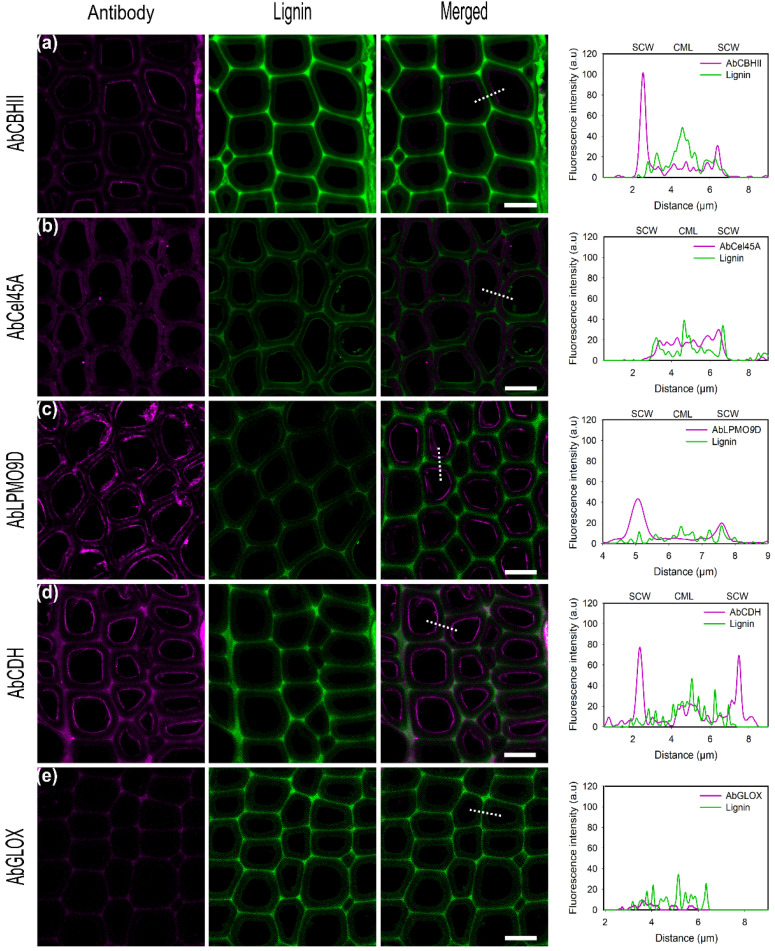
CLSM images of conjugated rabbit polyclonal antibody (**a**) AbCBHII, (**b**) AbCel45A, (**c**) AbLPMO9D, (**d**) AbCDH, (**e**) AbGLOXagainst *P. chrysosporium* enzymes on poplar cross-sections showing fiber cells. Enzyme fluorescence is colored in magenta. Lignin autofluorescence is shown in green. The merged column displays the overlay of enzyme and autofluorescence channels. Graphs showing overlay profile (dash line) of line scans over fiber cell wall, showing secondary cell walls (SCW) and compound middle lamella (CML). The brightness was enhanced to allow proper visualization using the same factor. Scale bars correspond to 10 µm.

To detect the secretion of enzymes by fungal hyphae, antibodies are necessary since the enzymes cannot be tagged directly in the samples. Therefore, the direct binding of conjugated fungal enzymes to the poplar cell walls and their indirect detection using conjugated antibodies were examined in this study. As previously described for α-cellulose as the substrate, and also for the experiments using conjugated specific antibodies, the image acquisition settings had to be adjusted to compensate for the weaker signal intensities (Table S3). In general, a good recognition of the target enzymes localized at the inner SCW was observed and similar results as those with directly labeled enzymes could be obtained (Figure 6). However, a higher unspecific binding to CML was also detected, resulting in a higher background. This could be explained by the unspecific antibody interaction with the cell wall matrix. To address the unspecific binding, usually a blocking step with BSA is performed before the incubation with the primary antibody. In our case, no blocking step was performed before the antibody incubation. However, 1% BSA was added to antibody solutions to reduce the possible unproductive binding and increase protein stability. In addition, Western blot and indirect ELISA titers indicated that all antibodies have different specificities and sensitivities towards their targeted antigens. AbCBHII showed high selectivity for CBHII in Western blot analysis (Figure 1) as no bands could be detected for the negative control. However, due to the low sensitivity and low labeling yield (Table 2), antibody dilution had to be reduced to 1:50. As observed for CBHII, higher fluorescence was detected in SCW than CML. Although unspecific binding was increased regarding the directly labeled enzyme (Figure 6a), AbCel45A could be observed binding homogeneously to the whole poplar cell wall including CML and SCW and antibody aggregates could be detected at SCW regions (Figure 6b). The same binding pattern was observed for the directly labeled Cel45A reinforcing the specificity and sensitivity of the antibody towards the enzyme observed in Western blot and ELISA assays (Figure 1, Table 2). AbLPMO9D recognized the enzyme preferentially in SCW regions rather than in CML (Figure 6c). Moreover, microtome sectioning damaged poplar cell walls, increasing biopolymer exposition and promoting enzyme binding. AbCDH (Figure 6d) as well as AbCBHII bind preferentially to SCW rather than in CML regions as detected previously for CDH. As observed for the directly labeled GLOX, no signal was detected at poplar cell walls and a minor unspecific binding of the antibody was detected (Figure 6e).

**Figure 7:**
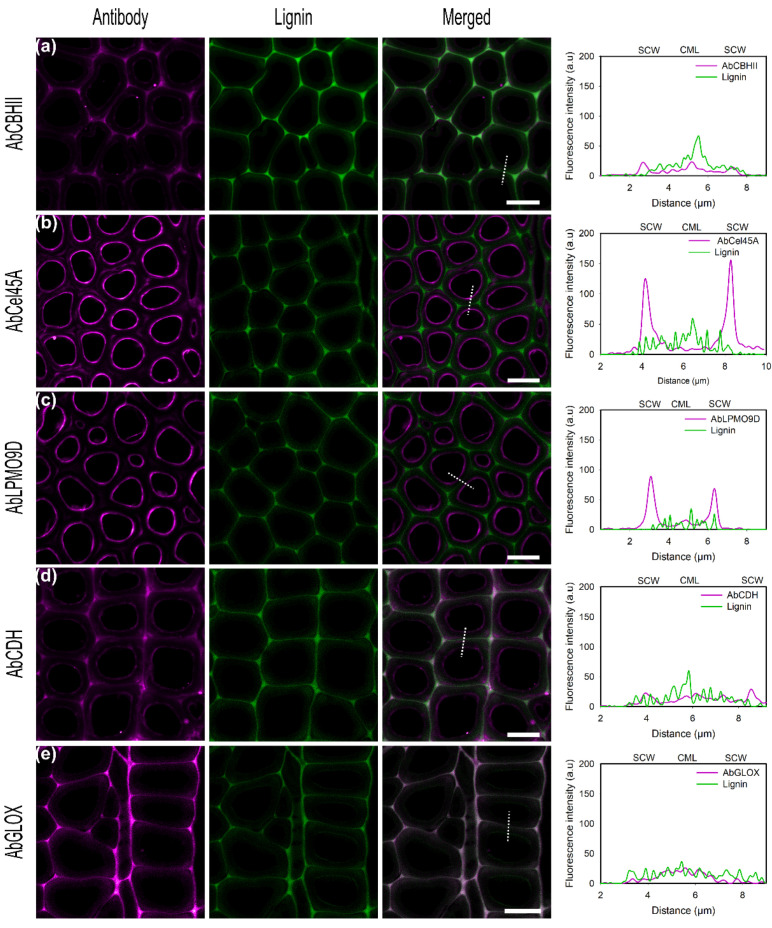
CLSM images of conjugated rabbit polyclonal antibody (**a**) AbCBHII, (**b**) AbCel45A, (**c**) AbLPMO9D, (**d**) AbCDH, (**e**) AbGLOX against *P. chrysosporium* enzymes on delignified poplar cross-sections showing fiber cells. Enzyme fluorescence is colored in magenta. Lignin autofluorescence is shown in green. The merged column displays the overlay of enzyme and autofluorescence channels. Graphs show the overlay profile (dash line) of line scans over the fiber cell wall, showing secondary cell walls (SCW) and compound middle lamella (CML). Scale bars correspond to 10 µm.

To test the effect of alkali pretreatment on enzyme recognition by the antibody, pretreated samples were incubated with enzymes and subsequently detected using conjugated antibodies (Figure 7). As observed for untreated poplar sections, conjugated antibodies localized fungal enzymes mainly at the inner cell wall regions as was also found with directly conjugated enzymes. In regard to AbCBHII (Figure 7a), no enhanced signal was detected after pretreatment and unspecific binding was also observed in lignin-rich areas. Nevertheless, the observed increase in unspecific binding agrees with the previous increase observed with directly labeled CBHII (Figure 4a). In contrast for AbCel45A, alkali pretreatment had a positive effect, as 5-fold higher fluorescence was detected on the SCW surface than on CML (Figure 7b). As observed for Cel45A, pretreatment enhances enzyme penetration into cell wall layers due to lignin and hemicellulose removal exposing cellulose macro-fibrils and microfibrils. After delignification, a higher signal localizing the enzyme in the same binding regions as for the directly labeled enzymes was observed for AbCBHII, AbLPMO9D, and AbCel45A. Interestingly, no increase of CDH loading on the pretreated sample, but rather a slightly lower surface binding of CDH, could be detected using fluorescently tagged AbCDH (Figure 7d).

Moreover, for AbGLOX, the same trend, as prior observed using the directly labeled GLOX, was found using the visualization based on specific antibodies. While no fluorescence signal was observed on the native biomass, a good signal was found on the pretreated wood sample that is in excellent agreement with the autofluorescence of lignin, emphasizing our observation that GLOX is potentially binding to the altered, and therefore exposed, lignin structure (Figure 7e).

## 3. Materials and Methods

### 3.1. Materials and Chemicals

All chemicals were of the highest purity grade available and were purchased from Sigma-Aldrich (St. Louis, MO, USA) unless stated differently. DyLight fluorescent dyes D550 and D633 complete kits for antibody labeling and maleimide-based thiol-coupling, as well as Pierce Dye Removal columns, were purchased from ThermoFisher (ThermoFisher Scientific Inc., Waltham, MA, USA). Endo Hf was obtained from New England Biolabs (Ipswich, MA, USA). Mini-PROTEAN TGX Stain-Free precast gels, Precision Plus Protein WesternC standard marker, and trans blot turbo pack 0.2 µm nitrocellulose membrane were obtained from Bio-Rad Laboratories (Hercules, CA, USA). Vivaspin 500 centrifugal concentrators were obtained from Sartorius (Göttingen, DE, USA), α-cellulose with a particle size of 125 µm was obtained from a commercially available preparation from Sigma (C8002). Microscope slides of 1 mm thickness were obtained from DWK Life Sciences (Wertheim, DE, USA) and coverslips #1 (0.13–0.16 mm) were purchased from Epredia-Fisher Scientific (Waltham, MA, USA).

### 3.2. Site-Directed Mutagenesis

The cDNA sequences encoding for hydrolases and oxidoreductases from *P. chrysosporium* strain CBS 481.73 with their native secretion signal were obtained from the GenBank database (https://www.ncbi.nlm.nih.gov/genbank, accessed on 3 March 2019) or the genomic database FungiDB (http://fungidb.org/fungidb, accessed on 17 March 2019). Protein structures were retrieved from the protein data bank (PDB; https://www.rcsb.org/, accessed on 10 April 2019), prepared by structural predictions using SWISS-MODEL (https://swissmodel.expasy.org/, accessed on 11 September 2021) or the AlphaFold deep learning network (https://alphafold.ebi.ac.uk/, accessed on 16 October 2021), and were evaluated using PyMol [69] (Table 1) to identify exposed amino acid residues on the protein surface or linker regions where a cysteine residue could be introduced for fluorescent tagging. Cysteine variants of selected fungal enzymes were generated by exchanging an amino acid on the protein surface not involved in substrate binding or catalysis (Table 1, Figures S1 and S2). The physicochemical properties of the enzymes were calculated using protein sequences and structures (Table 3). Theoretical mass, isoelectric point, and aliphatic index were computed by ProtParam [70] using the amino acid sequences without signal peptide predicted by SignalP 5.0 and were removed prior to further calculations [71]. Protein surface hydrophobicity and hydrophobic surface area (Å^2^) were computed using PDBparam [72] using protein structures if available, or homology models if unavailable. Total accessible surface area (Å^2^) and percent side hydrophobic area were computed using VADAR [73].

### 3.3. Heterologous Expression

*P. chrysosporium* strain CBS 481.73 was obtained from the Westerdijk Fungal Biodiversity Institute. Selected enzymes were recombinantly produced in *Pichia pastoris* and purified in accordance with published protocols: CBHII [74], Cel45A [28,75], LPMO9D [51], CDH [76], GLOX [77]. Further details on enzyme production and purification are described in Supplementary Method S1.

### 3.4. Enzyme Labeling

Heterologously produced hydrolases and oxidoreductases were directly labeled with DyLight D550 and D636 via maleimide coupling using the sulfhydryl-reactive dyes according to the instructions provided by the manufacturer. In brief, the labeling reactions were performed in 50 mM potassium phosphate buffer, pH 7.20, containing 150 mM sodium chloride with protein concentrations of 2–5 mg mL^−1^. A 5-fold molar excess of tris(2-carboxyethyl)phosphine (TCEP) was added and incubated at 21 °C for 20 min to reduce cysteine residues. Subsequently, a 7-fold excess of the respective dye was added and the labeling reaction was carried out at 4 °C for 3 days in the dark. Labeled enzymes were purified from an excess of dye using Pierce Dye Removal Columns according to the manufacturer’s protocol. The labeling efficiency was calculated by measuring UV/Vis absorbance spectra of the conjugated enzymes according to DyLight dye properties (Table S1, Figure S3). Conjugated enzymes were further concentrated using Vivaspin 500 centrifugal concentrators and buffer was exchanged to 50 mM phosphate buffer pH 6.50 and stored at −80 °C. To exclude possible interferences between fluorescent labeling and enzymatic properties, the enzymatic activity of conjugated and unconjugated CBHII, CDH and GLOX was evaluated using published procedures (Supplementary Method S2). Briefly, CBHII activity was tested by incubating the enzyme with carboxymethyl cellulose (CMC, 3% in water for 14 h at 40 °C), and the soluble products were analyzed on a Summit HPLC analytical system from Dionex (ThermoFisher Scientific) equipped with a P680 pump, an ASI-100 autosampler and a RI-101 detector (Shodex-Showa Denko America, Inc., New York, NY, USA) and were quantified using a calibration curve obtained with cellobiose. The catalytic activity of CDH was detected photometrically by monitoring 2,6-dichloroindophenol (DCIP) reduction at 520 nm (ε520 = 19.6 mM^−1^ cm^−1^) as previously described [78,79]. GLOX activity was measured spectrophotometrically by monitoring 2,2′-azino-bis(3-ethylbenzothiazoline-6-sulfonic acid) (ABTS) oxidation at 420 nm (ε420 = 36 mM^−1^ cm^−1^) in a horseradish peroxidase coupled assay as previously described [77,80].

### 3.5. Enzyme Preparation for Immunization

Two mg of purified enzymes were rebuffered to 100 mM phosphate buffer pH 6.0 using PD-10 desalting columns (GE Healthcare, Chicago, IL, USA) and deglycosylated using Endo Hf (NEB). The commercial Endo Hf with a concentration of 1,000,000 U mL^−1^ was diluted to a final concentration of 10 U mL^−1^ and deglycosylation was carried out for 18 h at 4 °C. Deglycosylated enzymes were used for the immunization protocol to avoid any interference of the N-glycosylation during antibody generation and were supplemented with 5% glycerol to improve conformational stability. In order to purify the generated antibodies by affinity chromatography, 2 mg of glycosylated enzymes in 100 mM phosphate buffer pH 6.0 supplemented with 5% glycerol were supplied to ThermoFisher Scientific in charge of producing custom-made polyclonal rabbit antibodies and their affinity purification.

### 3.6. Antiserum Preparation

Specific antibodies were produced in two New Zealand white rabbits (specific pathogen-free) by ThermoFisher (Rockford, IL, USA). Briefly, rabbits were immunized intradermally with a suspension of phosphate-buffered saline (PBS)/Complete Freund’s adjuvant containing 0.25 mg of antigen and were boosted after 14, 42, and 56 days with a suspension of PBS/Incomplete Freund’s adjuvant containing 0.10 mg of antigen. The immune serum was collected 90 days after the first immunization. Obtained antibodies were purified by affinity chromatography using the corresponding antigens and antibody titers were determined by indirect enzyme-linked immunosorbent assays (ELISA). Titers (given in ng mL^−1^) indicate the minimum concentration at which the purified antibodies can effectively detect the antigens. Finally, the purified antibodies were provided in PBS pH 7.4 supplemented with 0.05% sodium azide to prevent microbial growth. For long-term storage at −20 °C, glycerol was added to purified antibodies to a final concentration of 50%.

### 3.7. Antibody Labeling

Purified antibodies (Table 2) were labeled with DyLight dye D550 and D633 antibody labeling kits activated with an NHS ester moiety to react with exposed N-terminal α-amino groups or the ε-amino groups of lysine residues. Labeling reactions were performed in PBS pH 7.4 with antibody concentrations of 0.1–0.3 mg mL^−1^. Antibody solutions were mixed with 10-fold molar excess DyLight dyes and were vortexed gently to ensure complete mixing. Reaction tubes were covered with aluminum foil and were incubated at 21 °C for 1 h. Free dye was separated using 250 µL of Pierce dye removal columns resin. The performance of the labeling reaction was examined by measuring the absorbance of the labeled antibody at DyLight dye maximum absorbance wavelength (Figure S4) and the moles of dye per mole of protein were calculated according to DyLight dye properties (Table S1) following the manufacturer’s instructions.

### 3.8. Western Blotting

The specificity of purified antibodies was evaluated by Western blot analysis. First, the cultivation supernatant from *P. chrysosporium* culture after ~7 days of fermentation using microcrystalline cellulose as a carbon source was used as a negative control (−) and the purified set of enzymes was separated by Mini-PROTEAN TGX stain-free precast gels (Bio-Rad) according to the manufacturer’s guidelines. To determine the molecular weight, the Precision Plus Protein WesternC standard ladder (Bio-Rad) was used. The gel was blotted onto a trans blot turbo pack 0.2 µm nitrocellulose membrane using the transblot turbo-transfer system. The membrane was incubated with 1% (*w*/*v*) bovine serum albumin (BSA) in PBS pH 7.4 overnight at 4 °C to block unspecific antibody binding. Subsequently, the membrane was washed with PBS containing 0.1% (*v*/*v*) Tween 20 three times for 5 min each. Then, the membrane was incubated with diluted (1:5000) conjugated antibodies (stock solution 10 µM) in a blocking solution for 2 h at 21 °C. After three washes in PBS buffer containing 0.1% (*v*/*v*) Tween 20 for 5 min each, the antibody reaction was detected by exciting the membrane with UV transillumination using a Bio-Rad ChemiDocTM XRS+ Imaging system with Cy 2 (Emission 520 nm) and Qdot 625 (Emission 625 nm) fluorescence options to image conjugated antibodies with DyLight 550 and DyLight 633, respectively.

### 3.9. Substrate Preparation

Ten mg of α-cellulose, with a particle size of <125 µm, were added to 1 mL 100 mM sodium phosphate buffer, pH 6.50, to form a cellulose-suspension slurry. Poplar sections were obtained from a 5-year-old white poplar tree (*Populus alba*) harvested in Vienna, Austria. Small sample blocks were cut out from poplar branches, debarked, and treated at 80 °C in a water bath for 1 h to eliminate possible fungal and endogenous bacteria. Without any embedding routine, 7–12 µm-thick transverse sections were prepared on a sliding microtome (Microm HM 360, Waldorf, DE, USA) using stainless steel blades (MS200, Micros-Austria, St. Veit, AT, USA). To investigate the effect of a partial delignification on enzyme interaction, consecutive slices were used to avoid artifacts caused by natural heterogeneity. Samples were delignified using an aqueous solution of 10% KOH for 4 h at 80 °C. After incubation, sections were extensively rinsed with ultrapure water and stored at 4 °C for further analysis.

### 3.10. Enzyme Adsorption

Adsorption experiments were performed at 21 °C for 20 min, sufficient to ensure binding, and were protected from the light to avoid fluorescent quenching. For α-cellulose experiments, the stirred solution (1 mg mL^−1^) was mixed with 1 µM directly labeled enzyme to a final substrate concentration of 5 mg mL^−1^. The reaction was carried out in the dark with constant shaking (700 rpm) to prevent particles from settling and to promote enzyme binding. Particles were recovered at 10,000× *g* for 1 min. The supernatant with an unbound enzyme was then discarded and the pellet resuspended in 10 µL of 100 mM sodium phosphate buffer pH 6.50 and stored at 4 °C for further analysis. For antibody detection experiments, α-cellulose pellet was obtained after enzyme incubation was resuspended with primary conjugated antibodies (1.5 µM) diluted to 1:100 in PBS pH 7.40 containing 1% BSA for 2 h at 21 °C and constant shaking at 500 rpm protected from the light. The α-cellulose suspension was centrifuged 10,000× *g* for 1 min and was washed with PBS containing 0.1% (*v*/*v*) Tween 20 three times for 5 min each to remove unbound antibodies and to ensure minimal background fluorescence and a high signal-to-noise ratio and was stored at 4 °C for CLSM analysis. For adsorption experiments on poplar wood, 5 µL of 1 µM enzyme solutions were allowed to adsorb on 10 µm transverse sections for 20 min. The unbound enzyme was removed by carefully washing the wood section with buffer and was stored for subsequent visualization. For indirect detection, wood sections were incubated within diluted antibodies solutions (1:100) in PBS containing 1% BSA at 4 °C overnight with constant shaking of 500 rpm protected from the light. After incubation, wood sections were washed three times for 5 min each in PBS containing 0.1% (*v*/*v*) Tween 20 and stored at 4 °C.

### 3.11. Confocal Laser Scanning Microscopy (CLSM)

Poplar sections or α-cellulose fibers were mounted on glass slides (1 mm thickness, DWK Life Sciences) without coating with cover glasses #1 (0.13–0.16 mm from Epredia-Fisher scientific). CLSM imaging was performed on an inverted Leica SP8-STED (Leica Microsystems, Wetzar, DE, USA) single point scanning confocal commercial microscope attached to a fully motorized DMi8 microscope base and a Leica linear encoded stage at the Vienna Institute of Biotechnology (VIBT) Imaging Centre at BOKU. Images were acquired at room temperature using a Leica 63×/1.2NA HC PlanApo water immersion objective lens (with an objective magnification of 3.5) together with a Leica tunable WLL2 white light laser illumination AOBS modulated and Leica HyD2 detectors. Images were acquired sequentially by frame scanning unidirectional at 200 Hz using the galvanometer-based imaging mode, with a voxel size of 0.052 µm × 0.052 µm and a pixel dwell of 1.2 µs. The imaging area was set to 52.71 µm × 52.71 µm unless otherwise indicated (Tables S2 and S3) following Nyquist criteria calculated using the Nyquist Calculator app from Huygens (https://svi.nl/NyquistCalculator, accessed on 15 June 2021) in the Leica LASX Acquisition software (version 3.5.2.18963) and was saved as a LIF file. For all images, the pinhole size was 111.4 µm, calculated at 1 A.U for 580 emission. To reduce crosstalk, notch filters from Leica NF 514 and NF 488/561/633 nm were applied when collecting D500 and D633 emissions respectively. DyLight D550 was excited at 516 nm, with a White Light Laser (WLL) with an intensity of 15%, unless stated differently (Tables S2 and S3) and emissions were collected using a HyD2 detector without gaiting and 100% gain between 560–600 nm. For DyLight D633 fluorophore, the excitation wavelength used was 636 nm with a laser intensity of 15%. The emission was collected by a HyD2 detector with no gaiting, with a collection window of 660–700 nm and 100% gain. Poplar lignin autofluorescence was excited at 405 nm (with a laser power of CW 50 mW) and a 5% laser intensity. The emission was collected by a HyD detector between 415–550 nm. Channel crosstalk between lignin autofluorescence and DyLight dye channels was tested on poplar microtome slices (Figure S6). In all channels, line average and frame accumulation were adjusted to increase the fluorescence signal while preventing photobleaching, which was not observed at significant levels over the course of the experiments. For α-cellulose samples, lignin autofluorescence was not imaged. Z-stacks were acquired with a step size of 1 µm in a range between 5 µm and 9 µm (Table S2, Figures S7 and S8).

### 3.12. Data Processing

LAS X software (Leica, DE, USA) was employed to control the microscope. All confocal images were further deconvolved with Huygens Professional v.19.04 (Scientific Volume Imaging, NL, http://svi.nl, accessed on 17 September 2021). The point spread function (PSF) was theoretically calculated by the software. During deconvolution, the background was automatically estimated by using the lowest estimation mode and an area radius of 0.7 µm (Tables S2 and S3). Manual background estimations were performed according to the Huygens program instructions after image evaluation. As a deconvolution algorithm, the Classic Maximum Likelihood Estimation (CMLE) with a maximum of 40 iterations and a quality threshold of 0.05 was chosen. The signal-to-noise ratio (SNR) was evaluated for each image individually and was adjusted where necessary (Tables S2 and S3). The iteration mode, bleaching correction, and brick layout were set automatically by the software. Acquired images were evaluated using the open-source software FIJI [81]. Regions of interest (ROIs) were selected manually for two types of positions: the inner cell wall in the vicinity of the cell lumen (referred to as the secondary cell wall (SCW)), and compound middle lamella regions (CML) including cell corners. For each type of position, five ROIs were selected, and the mean values of the maximum intensities were transferred and evaluated using Sigma Plot software (Version 12.5, Systat Software, San Jose, CA, USA).

## 4. Conclusions

To fully understand enzymatic biomass deconstruction, it is important to elucidate the interaction of enzymes with the polymers forming the hierarchically structured plant cell wall. Visualization at the nano- and meso-scale is a starting point to investigate enzyme localization and interaction on the cell wall. In this study, CLSM has been used to localize enzymes by the conjugation with fluorescent dyes either directly linked to the enzymes of interest or with enzyme-specific polyclonal antibodies. Direct conjugation of fluorophores can only be used on engineered and recombinantly produced enzymes that can be studied alone or in combination on substrates. If their expression through a growing fungus is followed, only detection with antibodies is possible. For both methods, the same enzyme binding pattern, which has often been considered as a rate limiting step [82], was observed at the innermost layer of the secondary cell wall, showing the highest density of adsorbed enzymes, which is in good agreement with previous reports [49]. Nonetheless, the method employed in our study does not allow the distinction between enzymes that have been unspecifically adsorbed and those that have been productively bound. However, the signal intensities observed by CLSM for the indirect detection by labeled antibodies were lower, and slightly higher background signals were also detected, most likely caused by the unspecific binding of labeled antibodies to the biomass substrate. However, the application of secondary antibodies or other techniques available for the engineering and modification of antibodies could potentially improve signal intensities. Moreover, the presence of an antibody can interfere with the substrate binding of an enzyme as we found for CBHII, which is probably caused by the interaction of the antibody in regions of the enzyme that are crucial for substrate binding and therefore reduce its binding affinity. 

This study demonstrates the potential of CLSM using directly labeled enzymes as well as their detection by tagged antibodies in order to analyze the localization of lignocellulolytic enzymes on the plant cell wall. The verification that directly tagged enzymes, and indirectly via antibodies detected enzymes, give quite similar results, allowing a combination of studies with isolated enzymes and a comparison of results obtained with enzymes secreted by fungi during plant cell wall degradation.

## Figures and Tables

**Table 1:** Labeling efficiencies of heterologously produced hydrolases and oxidoreductases from *P. chrysosporium*. A single cysteine residue was introduced within the native protein structures. No crystal structure of GLOX is available. A homology model was built using the SWISS-MODEL with the crystal structure of related galactose oxidase (GAOX) from *Fusarium* sp. used as a template (pdb: 1GOF).

Enzymes	PDB	Cysteine Mutation	Conjugated DyLight Dye	Enzyme Recovery Yield (%)	Moles Dye/Moles Protein
CBHII	5XCZ	S72C	DL550	84.5	1.04
Cel45A	3X2N	C207	DL633	96.6	0.80
LPMO9D	4B5Q	S199C	DL633	77.1	0.14
CDH	1KDG	A770C	DL550	81.2	0.37
GLOX	1GOF*	S551C	DL550	71.9	0.28

**Table 2:** Labeling efficiencies of rabbit polyclonal antibodies produced against *P. chrysosporium* hydrolases and oxidoreductases.

Polyclonal Antibodies	ELISA Titer (ng mL^−1^)	Conjugated DyLight Dye	Antibody Recovery Yield (%)	Moles Dye/ Moles Protein
AbCBHII	625	DL550	73.1	1.86
AbCel45A	39	DL633	56.3	1.17
AbLPMO9D	156	DL633	78.6	3.79
AbCDH	78	DL550	78.7	0.94
AbGLOX	156	DL550	59.7	4.91

**Table 3:** Hydrolases and oxidoreductase physicochemical properties computed using protein sequences and protein structures. For GLOX, an AlphaFold prediction model was constructed from the protein sequence. N.D., not determined.

Enzyme	Uniprot KB	M (g mol^−1^)	Hydrophobic Accessible Area (Å^2^)	% Side Accessible Hydrophobic Area	pI	Experimental pI	Reference pI
CBHII	Q02321	46,391	4058	33.23	4.92	4.87	[50]
Cel45A	B3Y002	18,984	1602	24.91	5.38	N.D.	-
LPMO9D	H1AE14	23,502	1704	21.13	4.54	4.8	[51]
CDH	Q01738	79,747	2074	27.49	5.12	4.2	[52]
GLOX	Q01772	54,532		35.34	5.18	N.D.	-

## Data Availability

All data are reported in the manuscript or the supplementary materials.

## Supplementary Materials

The following supporting information can be downloaded at: www.mdpi.com/article/10.3390/ijms23095216/s1. [28,51,75,76,77,78,79,80,83,84].
