# Supplementary material for: Fluorescent Imaging of Extracellular Fungal Enzymes Bound onto Plant Cell Walls
Source: Int J Mol Sci. 2022 May 6;23(9):5216. doi: 10.3390/ijms23095216 (PMC9105846; doi:10.3390/ijms23095216)
Supplement: Supplementary file 1 [file ijms-23-05216-s001.zip › ijms-1696175-supplementary.pdf]

Supplementary Materials

Fluorescent imaging of extracellular fungal enzymes bound onto plant cell walls

Neus Gacias-Amengual [1], Lena Wohlschlager [1], Florian Csarman [1]*, Roland Ludwig [1]

[1] University of Natural Resources and Life Sciences, Vienna (BOKU), Department of Food Science and Technology, Institute for Food Technology, Biocatalysis and Biosensing Laboratory, Muthgasse 18, 1190 Vienna, Austria; neus.gacias@boku.ac.at (G.N), lena@wohlschlager.at (W.L), roland.ludwig@boku.ac.at (L.R)
* Correspondence: florian.csarman@boku.ac.at (C.F)

CBHII S72C
UniProtKB: Q02321

```
MKSTAFFAALVTLLPAYVAGQASEWGQCGGIGWTGPTTCVSGTTCTVLNPYYSQCLPGSAVTTTSVITSHSCSVSSVSSHSGSSTS
TSSPTGPTGTNPPPPPSANNPWTGFQIFLSPYYANEVAAAAKQITDPTLSSKAASVANIPTFTWLDSVAKIPDLGTYLASASALGK
STGTKQLVQIVIYDLPDRDCAAKASNGEFSIANNGQANYENYIDQIVAQIQQFPDVRVVAVIEPDSLANLVTNLNVQKCANAKTTY
LACVNYALTNLAKVGVYMYMDAGHAGWLGWPANLSPAAQLFTQVWQNAGKSPFIKGLATNVANYNALQAASPDPITQGNPNYDEIH
YINALAPLLQQAGWDATFIVDQGRSGVQNIRQQWGDWCNIKGAGFGTRPTTNTGSQFIDSIVWVKPGGECDGTSNSSSPRYDSTCS
LPDAAQPAPEAGTWFQAYFQTLVSAANPPL
```

Cel45A C207 (added to C-terminus)
UniProtKB: B3Y002

```
MAKLSMFLGFVAVATLASALTVSEKRATGGYVQQATGQASFTMYSGCGSPACGKAASGFTAAINQLAFGSAPGLGAGDACGRCFAL
TGNHDPYSPNYTGPFGQTIVVKVTDLCPVQGNQEFCGQTTSNPTNQHGMPFHFDICEDTGGSAKFFPSGHGALTGTFTEVSCSQWS
GSDGGQLWNGACLSGETAPNWPSTACGNKGTAPSC
```

LPMO9D S199C
UniProtKB: H1AE14

```
MKAFFAVLAVVSAPFVLGHYTFPDFIEPSGTVTGDWVYVRETQNHYSNGPVTDVTSPEFRCYELDLQNTAGQTQTATVSAGDTVGF
KANSAIYHPGYLDVMMSPASPAANSPEAGTGQTWFKIYEEKPQFENGQLVFDTTQQEVTFTIPKSLPSGQYLLRIEQIALHVASSY
GGAQFYIG CAQLNVENGGNGTPGPLVCIPGVYTGYEPGILINIYNLPKNFTGYPAPGPAVWQG
```

CDH A770C
UniProtKB: Q01738

```
MLGRSLLALLPFVGLAFSQSASQFTDPTTGFQFTGITDPVHDVTYGFVFPPLATSGAQSTEFIGEVVAPIASKWIGIALGGAMNND
LLLVAWANGNQIVSSTRWATGYVQPTAYTGTATLTTLPETTINSTHWKWVFRCQGCTEWNNGGGIDVTSQGVLAWAFSNVAVDDPS
DPQSTFSEHTDFGFFGIDYSTAHSANYQNYLNGDSGNPTTTSTKPTSTSSSVTTGPTVSATPYDYIIVGAGPGGIIAADRLSEAGK
KVLLLERGGPSTKQTGGTYVAPWATSSGLTKFDIPGLFESLFTDSNPFWWCKDITVFAGCLVGGGTSVNGALYWYPNDGDFSSSVG
WPSSWTNHAPYTSKLSSRLPSTDHPSTDGQRYLEQSFNVVSQLLKGQGYNQATINDNPNYKDHVFGYSAFDFLNGKRAGPVATYLQ
TALARPNFTFKTNVMVSNVVRNGSQILGVQTNDPTLGPNGFIPVTPKGRVILSAGAFGTSRILFQSGIGPTDMIQTVQSNPTAAAA
LPPQNQWINLPVGMNAQDNPSINLVFTHPSIDAYENWADVWSNPRPADAAQYLANQSGVFAGASPKLNFWRAYSGSDGFTRYAQGT
VRPGAASVNSSLPYNASQIFTITVYLSTGIQSRGRIGIDAALRGTVLTPPWLVNPVDKTVLLQALHDVVSNIGSIPGLTMITPDVT
QTLEEYVDAYDPATMNSNHWVSSTTIGSSPQSAVVDSNVKVFGTNNLFIVDAGIIPHLPTGNPQGTLMSAAEQAAAKILALCGGP
```

GLOX S551C
UniProtKB: Q01772

```
MLSLLAVVSLAAATLAAPAASDAPGWRFDLKPNLSGIVALEAIVVNSSLVVIFDRATGDQPLKINGESTWGALWDLDTSTVRPLSV
LTDSFCASGALLSNGTMVSMGGTPGGTGGDVAAPPGNQAIRIFEPCASPSGDGCTLFEDPATVHLLEERWYPSSVRIFDGSLMIIG
GSHVLTPFYNVDPANSFEFFPSKEQTPRPSAFLERSLPANLFPRAFALPDGTVFIVANNQSIIYDIEKNTETILPDIPNGVRVTNP
IDGSAILLPLSPPDFIPEVLVCGGSTADTSLPSTSLSSQHPATSQCSRIKLTPEGIKAGWQVEHMLEARMMPELVHVPNGQILITN
GAGTGFAALSAVADPVGNSNADHPVLTPSLYTPDAPLGKRISNAGMPTTTIPRMYHSTVTLTQQGNFFIGGNNPNMNFTPPGTPGI
KFPSELRIETLDPPFMFRSRPALLTMPEKLKFGQKVTVPITIPSDLKASKVQVALMDLGFSSHAFHSSARLVFMESSISADRKSLT
FTAPPNGRVFPPGPAVVFLTIDDVTSPGERVMMGCGNPPPTLE
```

Figure S1. Introduction of a surface-exposed cysteine residue in hydrolase and oxidoreductase sequences for the maleimide coupling of the fluorescent dyes DyLight D550 and D633. The introduced cysteine residues are highlighted in red.

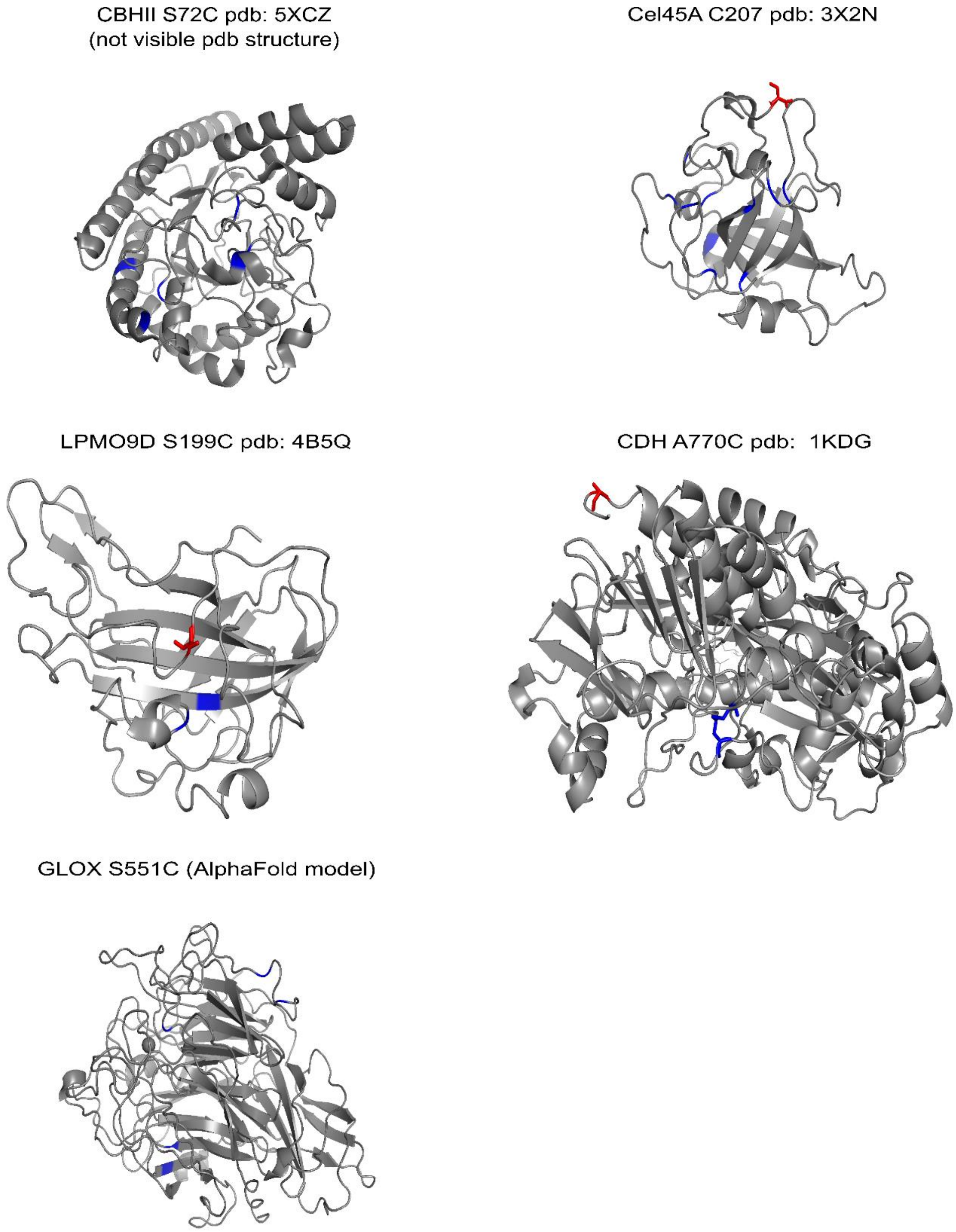

Figure S2. Protein structures of hydrolases and oxidoreductases showing the introduced cysteine residue for maleimide coupling. Deposited crystal structures and an AlphaFold model are used to show the introduced cysteine residues in red (and the native cysteine residues in blue).

Supplementary Method S1. Heterologous expression and purification of *P. chrysosporium* hydrolases and oxidoreductases**.**

Production and purification of selected enzymes was performed as described in previous studies CBHII [1], Cel45A [2,3], LPMO9D [4], CDH [5], and GLOX [6]. Briefly, hydrolase and oxidoreductase genes containing the native signal peptides were synthesized by BioCat GmbH (Heidelberg, DE) and cloned into the commercially available plasmid pPICZA (Invitrogen, Carlsbad, CA, USA) for the expression in the yeast Pichia pastoris. Plasmids were linearized with SacI-HF (New England Biolabs, Ipswich, MA, USA) according to NEB's Double Digest Protocol before transformation of P. pastoris. Electroporation and selection of transformants were carried out according to the EasySelectTM Pichia expression kit instruction manual (Invitrogen Ltd.). The recombinant proteins were produced using 5-L fermenters according to the Pichia Fermentation Process Guidelines (Invitrogen) using a BioFlo® 120 bioreactor (Eppendorf, Hamburg, DE). After 4 days of methanol-fed batch cultivation, the cells were removed by centrifugation and the supernatant was concentrated using a MiniKros Plus TFF filtration system (Repligen, Waltham, MA, USA) with a modified polyethersulfone membrane (mPES) and a molecular weight cut off (MWCO) of 10 kDa. The concentrated supernatant was fractionated on a Phenyl-Sepharose FF column (250 mL, GE Healthcare, Chicago, IL, USA) equilibrated with a 200 mM sodium acetate buffer containing 1 M ammonium sulfate (pH 5.0). The concentrated supernatant was supplemented with ammonium sulfate to a final concentration of 1 M and loaded to the column at room temperature. Fractions with the highest specific enzyme activity were pooled and concentrated using Vivaspin sample concentrators with 10 kDa MWCO and rebuffered to 20 mM potassium phosphate buffer (pH 7.0). Proteins were further purified by anion exchange chromatography using a Source 15Q (19 mL, GE Healthcare) equilibrated with 20 mM potassium phosphate buffer (pH 7.0) and the protein of interest was eluted in a linear gradient from 0 to 1 M NaCl in the same buffer. Finally, enzyme purity was verified by SDS-PAGE.

Supplementary Method S2. Activity assays performed with fluorescent dye-tagged CBHII, CDH, and GLOX.

Labeled and unlabeled CBHII (20 μg) was incubated with carboxymethyl cellulose (CMC, 3 % in water) for 14 h at 40 °C with shaking at 1200 rpm. The enzyme was inactivated by heating to 99 °C for 5 min. A blank consisting of the substrate and buffer instead of enzyme solution was treated the same way. The samples were applied to analysis on a Summit HPLC analytical system from Dionex (Thermo Scientific, Waltham, MA, USA) equipped with a P680 pump, an ASI-100 autosampler, and a RI-101 detector (Shodex). Separation was achieved on an Aminex HPX-87K column (Bio-Rad, Hercules, CA, USA) by isocratic elution with HQ-H_2O at 80 °C with a flow rate of 0.5 mL min^{-1}. For quantification, cellobiose standards were used and a calibration curve was constructed to determine the cellobiose release for unlabeled and labeled CBHII.

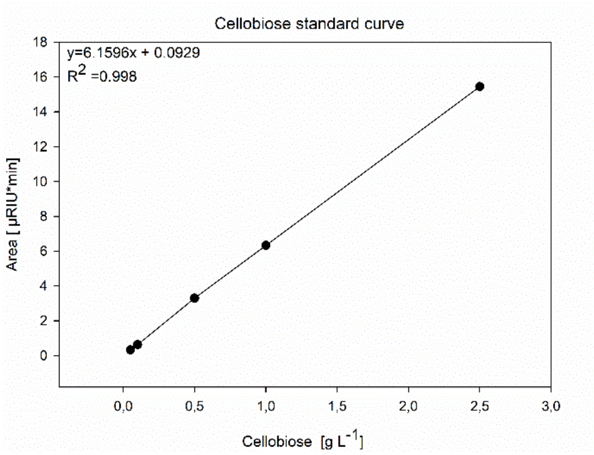

CBHII (20 μg)	Area [μRIU*min]	Cellobiose [g L^{-1}]
**unlabeled**	0.6380	0.089
**DyLight 550**	0.5857	0.080
**blank**	0.0000	0.000

The catalytic activity of CDH was detected photometrically by monitoring 2,6-dichloroindophenol (DCIP, $\varepsilon 550$ = 19.6 mM^{-1} cm^{-1}) reduction at 520 nm. The assay was performed in 100 mM sodium acetate buffer (pH 4.5) containing 30 mM lactose as substrate and 0.3 mM DCIP as electron acceptor. The reaction was followed for 180 s at 30 °C and the decrease of the absorption at 520 nm was recorded [7,8]. For GLOX, the catalytic activity was detected photometrically by monitoring 2,2′-Azino-di(3-ethylbenzthiazoline-6-sulfonic acid) (ABTS, $\varepsilon 420$ = 36 mM^{-1} cm^{-1}) oxidation at 420 nm using a coupled assay with horseradish peroxidase (HRP). The reaction was performed in 50 mM sodium-2,2-dimethylsuccinate buffer (pH 6.0) and contained 20 μL properly diluted enzyme solution, 1 mM ABTS, 7 units HRP and 10 mM methylglyoxal as substrate. The enzyme solution was incubated with 10 μL of 500 μM H_2O_2 in the pre-heated buffer for 5 minutes to activate GLOX, followed by the addition of the ABTS/HRP reagent and another incubation in the water bath for 5 min to set the assay temperature to 30 °C. The reaction was started by the addition of the substrate and followed for 180 s at 30 °C. GLOX activity assay was slightly modified from [9] as explained in detail before [6].

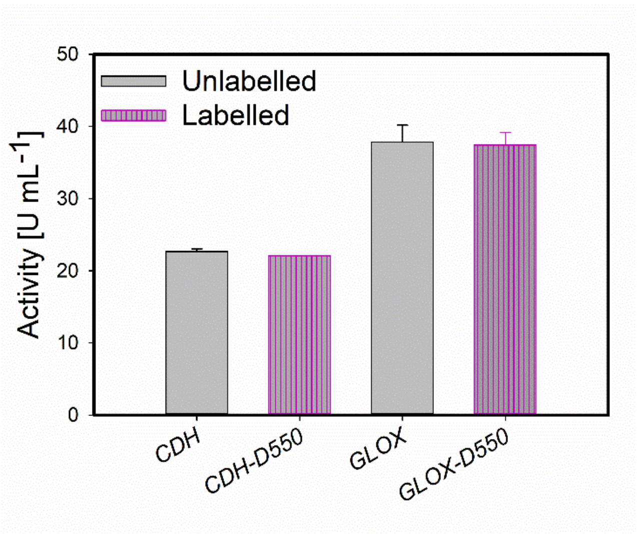

Unlabelled
Labelled
Activity [U mL-1]
50
40
30
20
10
0
CDH
CDH-D550
GLOX
GLOX-D550

Supplementary Method S3. To identify epitope regions on the antigens, three in-silico B-cell epitope prediction tools were used. The first relies on the antigen sequences, BepiPred-2.0 (https://services.healthtech.dtu.dk/service.php?BepiPred-2.0), DiscoTope-2.0 predicts discontinuous epitopes using antigen structures (http://tools.iedb.org/discotope/) and EpiPred (http://opig.stats.ox.ac.uk/webapps/sabpred) which uses geometric matching of the antibody and antigen protein structures. The structure of a monoclonal antibody (pdb: 1IGT) was used as a template for the EpiPred prediction tool.

The protein sequence of CBHII (UniProtKB: Q02321) was computed using BepiPred-2.0 with the default threshold value (0.5). The results predicted 12 continuous CBHII epitopes located at the CBM domain (16–28 aa, 34–39 aa amino acids), as well as residues forming the linker region (53–106 aa) and several regions on the catalytic domain (106–143, 154–160, 170–174, 194–211, 243–253,304-308,323–342, 370–401,413–443 aa). We used CBHII available structure (pdb: 5XCY). However, the crystal structure only represents the catalytic domain. DiscoTope-2.0 predicted epitopes are shown in yellow and EpiPred predicted epitope patches are ranked according to how likely they correspond to antibody recognition sites and highlighted in red.

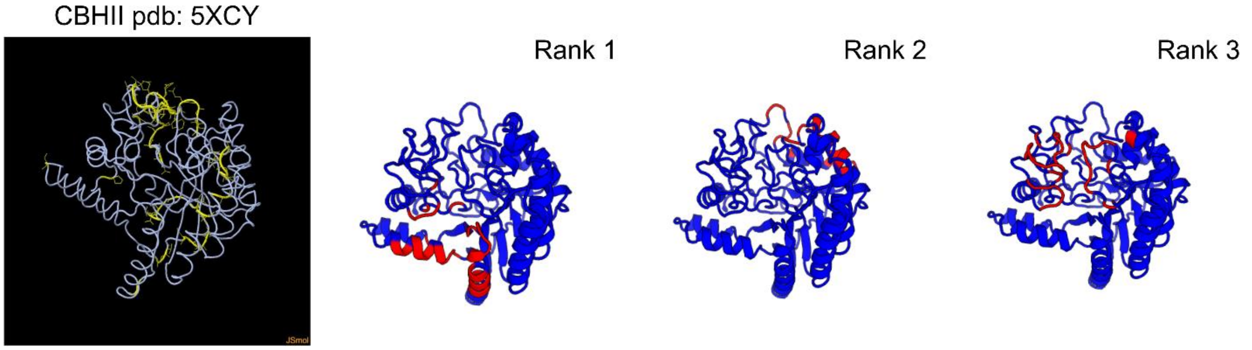

The analysis of Cel45A (UniProtKB: B3Y002) using BepiPred-2.0 predicted 6 possible epitope regions: 22–48 aa, 69–79 aa, 90–102 aa, 117–136 aa, 149–154 aa, 169–180 aa. Cel45A available structure (pdb: 3X2N) was used to predict the discontinuous epitopes by DiscoTope-2.0 and EpiPred. DiscoTope-2.0 predicted epitopes are shown in yellow and EpiPred predicted epitope patches are ranked according to how likely they correspond to antibody recognition sites and highlighted in red. For both structural models, epitopes were predicted at the edges of the substrate-binding cleft.

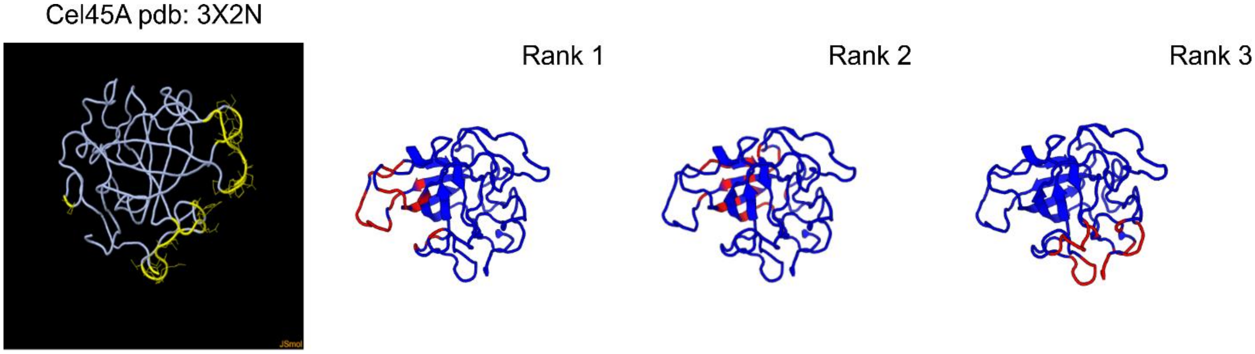

The analysis of LPMO9D (UniProtKB: H1AE14) using BepiPred-2.0 predicted 8 possible epitope regions: 31–33 aa, 40–54 aa, 62–74 aa, 107–119 aa, 126–141 aa, 152–154 aa,187–208 aa, 217–231 aa. LPMO9D available

structure (pdb: 4B5Q) was used to predict the discontinuous epitopes by DiscoTope-2.0 and EpiPred. DiscoTope-2.0 predicted epitopes are shown in yellow and EpiPred predicted epitope patches are ranked according to how likely they correspond to antibody recognition sites and highlighted in red. For both structural models, epitopes were predicted at LPMO9D loops involved in substrate recognition and binding (Wu et al. 2013).

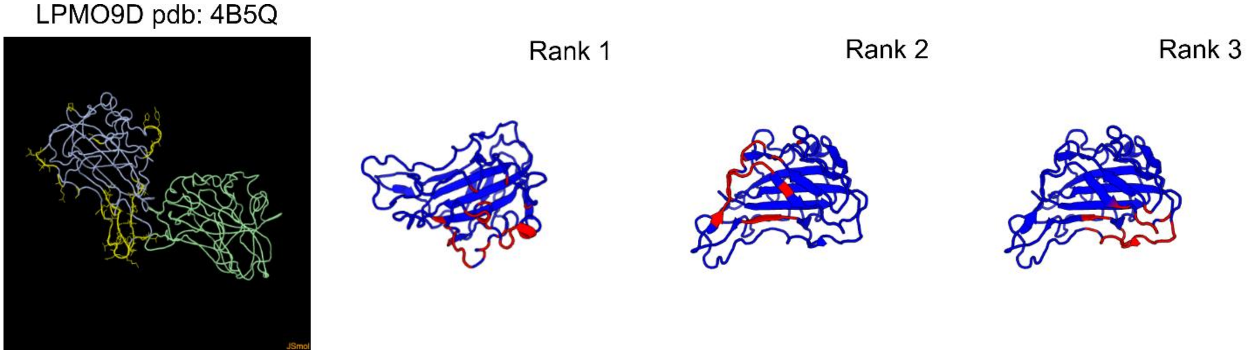

BepiPred-2.0 predicted 18 possible epitope regions on CDH sequence containing both domains: the b-type cytochrome (CYT) and flavodehydrogenase (DH) (UniProtKB: Q01738): 19–28 aa, 53–59 aa, 107–118 aa, 142–155 aa, 166–183 aa, 191–233 aa, 267–276 aa, 285–311 aa, 337–351 aa, 363–382 aa, 391–405 aa, 464–470 aa, 502–530 aa, 547–561 aa, 572–578 aa, 587–618 aa, 654–658 aa, 678–698 aa. The crystal structure of the flavodehydrogenase domain (DH) (pdb: 1KDG) was used to predict the discontinuous epitopes by DiscoTope-2.0 and EpiPred. Both methods predicted epitopes on the loop-and-lid region a hotspot for insertions and deletions among the GMC oxidoreductases [10].

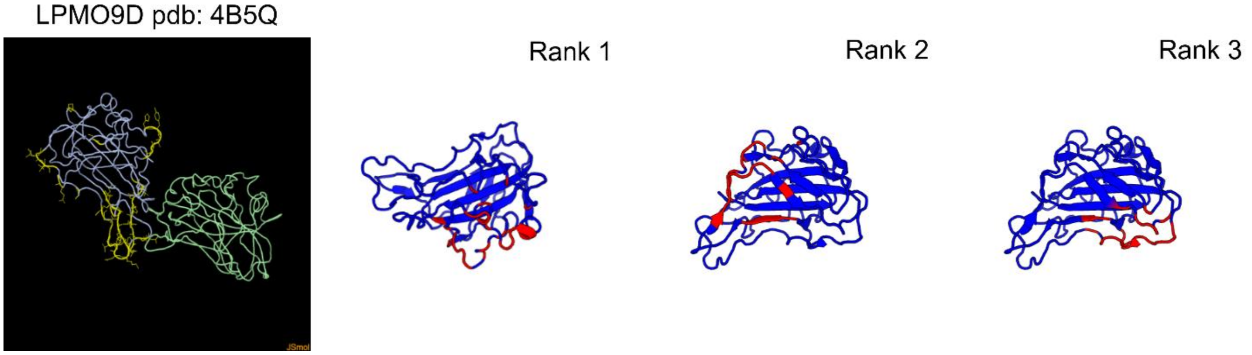

BepiPred-2.0 predicted 15 possible epitope regions on GLOX sequence (UniProtKB: Q01772): 17–34 aa, 57–68 aa, 108–123 aa, 132–154 aa, 178–185 aa, 195–212 aa, 238–257 aa, 285–305 aa, 308–326 aa, 349–367 aa, 381–395 aa, 420–464 aa, 473–479 aa, 508–513 aa, 554–556 aa. No crystal structure is available for GLOX, therefore a homology model was used to predict the epitopes using GaOX structure as a template (pdb: 1GOF) by DiscoTope-2.0 and EpiPred.

GLOX homology model pdb: 1GOF

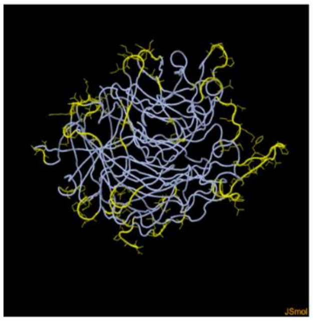

Rank 1

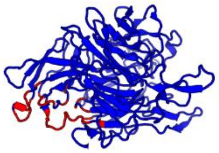

Rank 2

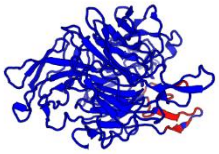

Rank 3

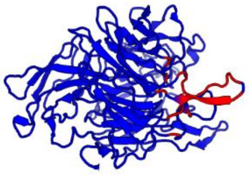

Table S1. Properties of the Thermo Scientific DyLight NHS-Ester Dyes. Abbreviations: Ex/Em Excitation and emission maxima in nanometers; Amax, maximum absorption in nanometers; ε, molar absorption coefficient at peak maximum (M-1 cm-1); CF, a correction factor (A280/Amax).

DyLight Dye	Ex/Em	A_{max}	ε	MW [g mol^{-1}]	CF
550	562/576	557	150,000	1040	0.081
633	638/658	627	170,000	1066	0.110

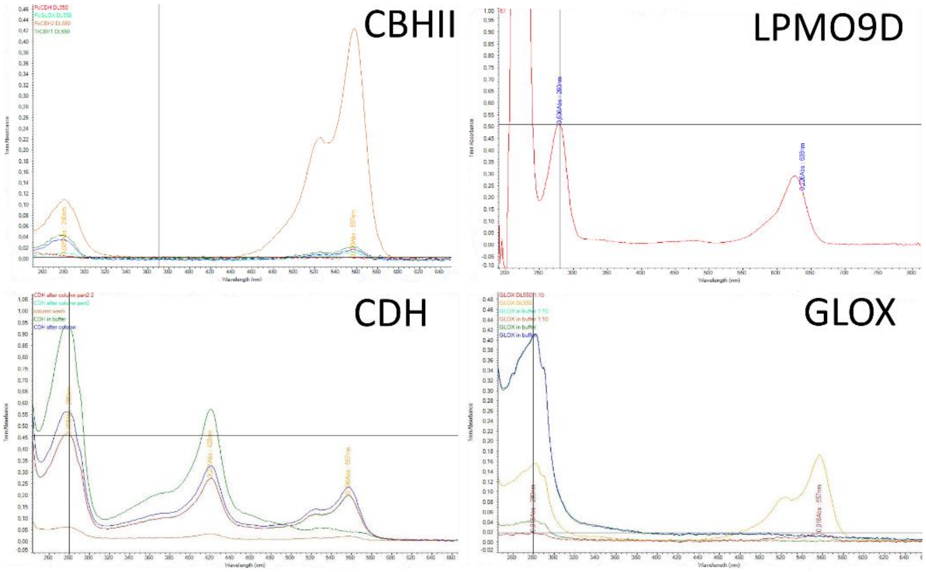

Figure S3. Spectra obtained for enzyme labeling using DyLight dyes D550 and D636. The maximum emission for D550 is at 557 nm and for D636 at 627 nm according to manufacturer's guidelines.

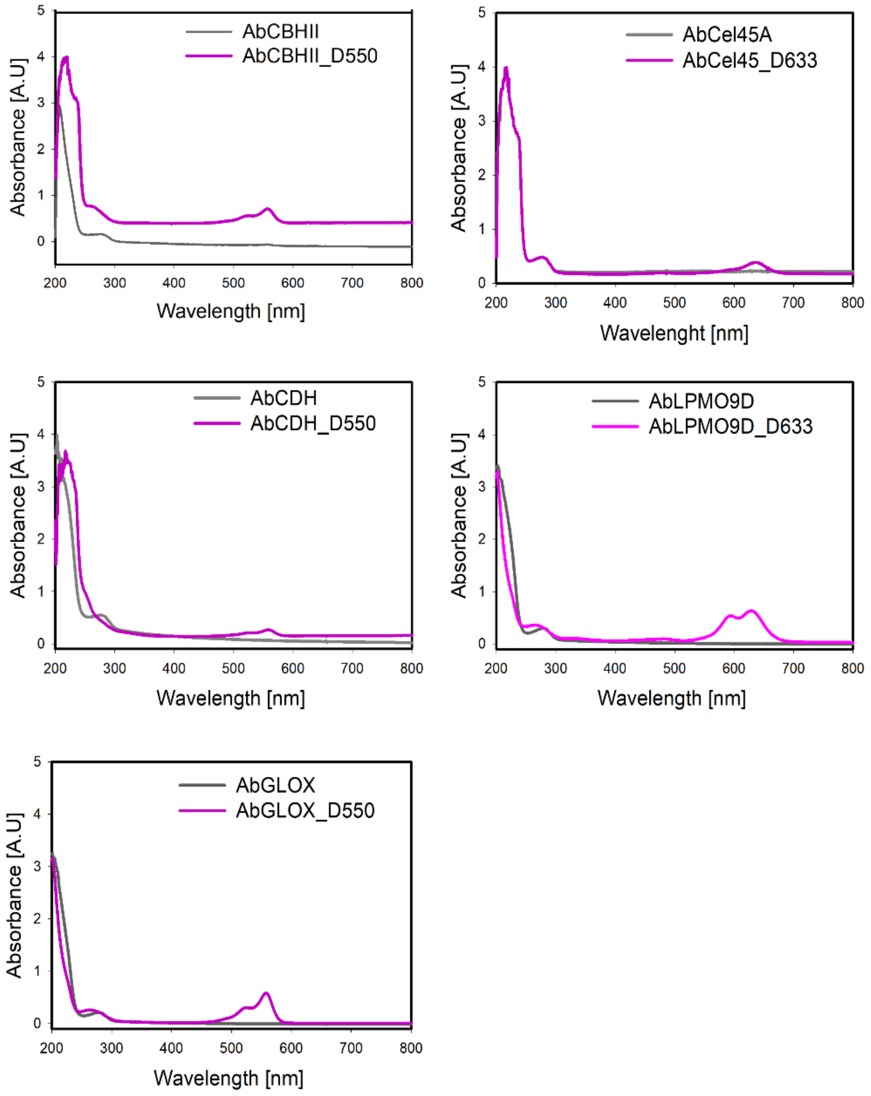

Figure S4. Spectra obtained for rabbit polyclonal antibody labeling using DyLight dyes D550 and D636. The maximum emission for D550 is at 557 nm and for D636 at 627 nm according to the manufacturer's guidelines.

Table S2. CLSM acquisition settings for directly labeled enzymes and labeled antibodies on α-cellulose adsorption studies. Abbreviations: Lint, laser intensity; LA, line average; FA, frame accumulation; Back, background subtracted using Huygens.*Background values computed manually; Iter, number of algorithm iterations; SNR, signal to noise ratio; Stop crit, algorithm stop criterion; N.D., not determined.

					DyLight Dye channel					Deconvolution DyLight channel			
α-cellulose	X (µm)	Y (µm)	Z (µm)	PTM	DyLight	LInt	HyD Gain	LA	FA	Back	Iter	SNR	Stop criterion
CBHII	52.79	52.79	7	280	D550	20	100	2	2	0.13	100	20	0.05
Cel45A	66.60	66.60	7	350	D633	10	80	2	1	0.78	100	20	0.05
LPMO9D	52.79	52.79	9	365	D633	15	100	2	2	1.65	100	20	0.05
CDH	52.78	52.78	5	300	D550	20	100	2	4	0.22	100	20	0.05
GLOX	52.71	52.71	8	300	D550	20	100	2	4	N.D.			
AbCBHII	52.53	52.53	7	240	D550	45	100	2	4	2	40	5	0.05
AbCel45A	52.96	52.96	7	320	D633	15	100	2	2	0.34	100	20	0.05
AbLPMO9D	52.79	52.79	6	320	D633	15	100	2	4	4.59	100	20	0.05
AbCDH	52.71	52.71	6	280	D550	20	100	2	4	0.88	100	20	0.05
AbGLOX	52.71	52.71	7	320	D550	20	100	2	4	N.D.			

Table S3: CLSM acquisition settings for directly labeled enzymes and labeled antibodies adsorption studies on poplar wood and alkali-treated wood. Abbreviations: Lint, laser intensity; LA, line average; FA, frame accumulation; Back, background subtracted using Huygens.*Background values computed manually; Iter, number of algorithm iterations; SNR, signal to noise ratio; Stop crit, algorithm stop criterion.

				DyLight Dye channel					Lignin autofluorescence channel				Deconvolution DyLight channel				Deconvolution autofluorescence			
	X (µm)	Y (µm)	PTM	DyLight	LInt	HyD Gain	LA	FA	LInt	HyD Gain	LA	FA	Back	Iter	SNR	Stop crit	Back	Iter	SNR	Stop crit
CBHII wood	52.79	52.79	280	D550	15	100	2	4	5	100	2	1	4	40	10	0.05	0.05	40	40	0.05
Cel45A wood	52.71	52.71	365	D633	10	80	2	2	5	100	2	1	8*	40	25	0.05	0.09	40	40	0.05
LPMO9D wood	52.71	52.71	320	D633	20	100	2	4	5	100	2	1	2.08	40	25	0.05	0.05	40	40	0.05
CDH wood	52.71	52.71	280	D550	20	100	2	2	5	100	2	1	1*	40	30	0.05	0.03	40	40	0.05
GLOX wood	52.88	52.88	280	D550	20	100	2	2	5	100	2	1	0.017	40	20	0.05	0.04	40	40	0.05
CBHII Alk	52.71	52.71	280	D550	15	100	2	4	5	60	2	1	25*	40	20	0.05	0.16	40	40	0.05
Cel45A Alk	52.62	52.62	320	d633	15	100	2	2	5	60	2	1	15*	40	35	0.05	0.09	100	20	0.01
LPMO9D Alk	52.79	52.79	320	D633	20	100	2	4	5	60	2	1	10*	40	20	0.05	0.05	40	40	0.05
CDH Alk	52.71	52.71	280	D550	20	100	2	2	5	60	2	1	10*	40	7	0.05	0.05	40	40	0.05
GLOX Alk	52.79	52.79	280	D550	15	100	2	4	5	60	2	1	1.5*	40	20	0.05	0.07	40	40	0.05
CBHII 1% BSA	52.65	52.65	300	D550	15	100	2	4	5	60	2	1	5	40	15	0.05	0.05	40	40	0.05
Cel45A 1%BSA	52.57	52.57	410	D633	10	80	2	2	5	60	2	1	3	40	30	0.05	0.04	40	40	0.05
GLOX 1% BSA	52.74	52.74	300	D550	20	100	2	2	5	60	2	1	1	40	20	0.05	0.05	40	40	0.05
AbCBHII	52.71	52.71	230	D550	45	100	2	3	5	60	2	1	0.39	40	10	0.05	0.01	40	40	0.05
AbCel45A	52.79	52.79	317	D633	20	100	2	2	5	100	2	1	6.48	40	10	0.05	0.07	40	40	0.05
AbLPMO9D	52.78	52.78	300	D633	15	100	2	4	5	100	2	1	1.62	40	20	0.05	0.14	40	30	0.05
AbCDH	52.78	52.78	280	D550	45	100	4	2	5	100	2	1	0.8	40	20	0.05	0.09	40	30	0.05
AbGLOX	52.71	52.71	317	D550	15	100	2	2	5	100	2	1	0.25	40	10	0.05	0.05	40	40	0.05
AbCBHII Alk	52.62	52.62	257	D550	45	100	2	4	5	60	1	1	2.98	40	20	0.05	0.12	40	40	0.05
AbCel45A Alk	52.79	52.79	392	D633	10	100	2	1	5	100	2	1	0.53	40	40	0.05	0.07	40	40	0.05
AbLPMO9D Alk	52.78	52.78	340	D633	15	100	2	2	5	100	2	1	0.3	40	40	0.05	0.05	40	40	0.05
AbCDH Alk	52.62	52.62	250	D550	45	100	2	2	5	100	2	1	0.27	40	20	0.05	0.05	40	40	0.05
AbGLOX Alk	46.89	46.89	280	D550	15	100	2	2	5	100	2	1	0.05	40	20	0.05	0.06	40	40	0.05

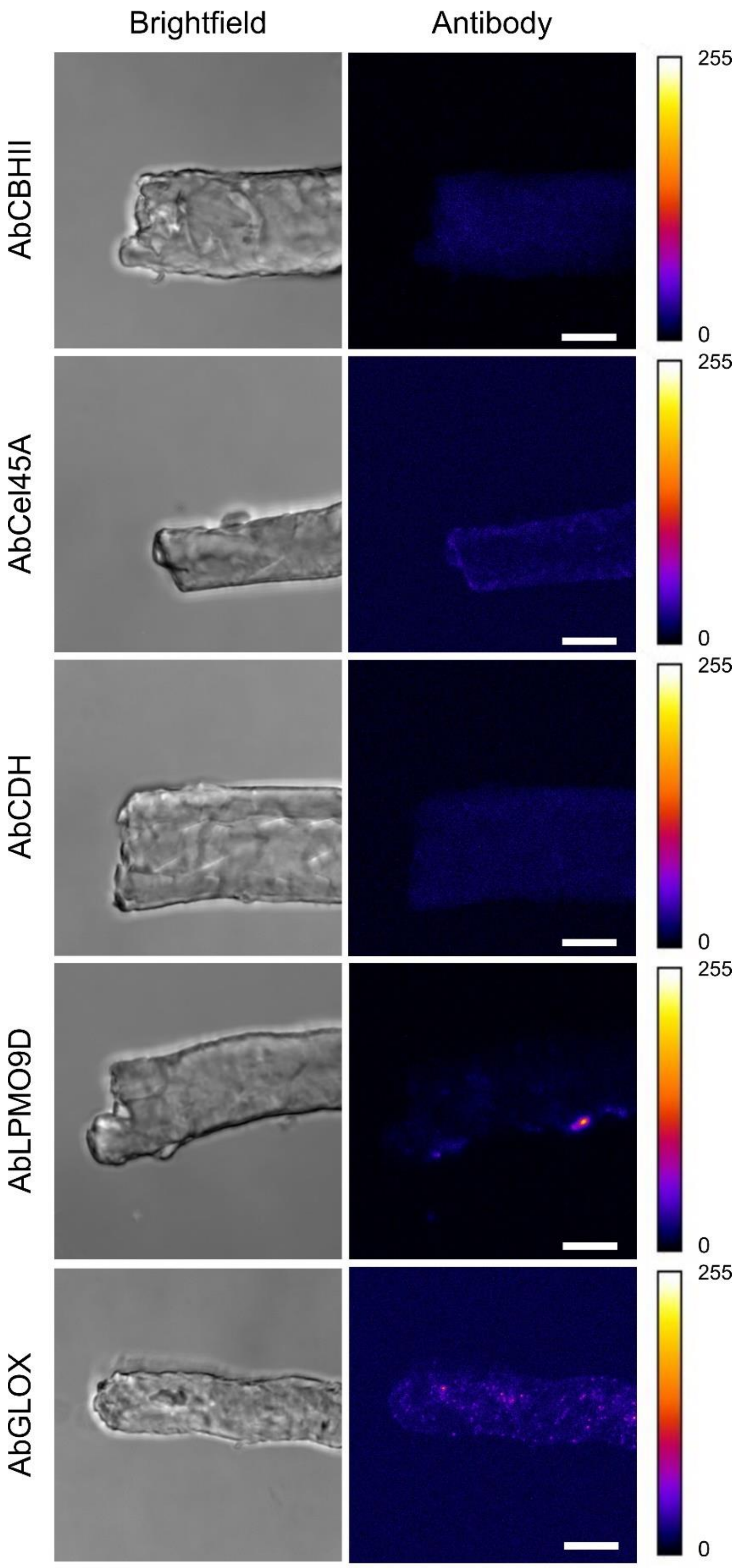

Figure S5. Antibody adsorption on α-cellulose. Antibodies were incubated for 2 h with α-cellulose fibers without the addition of the respective enzymes. Images were not deconvolved. Scale bars correspond to 10 µm.

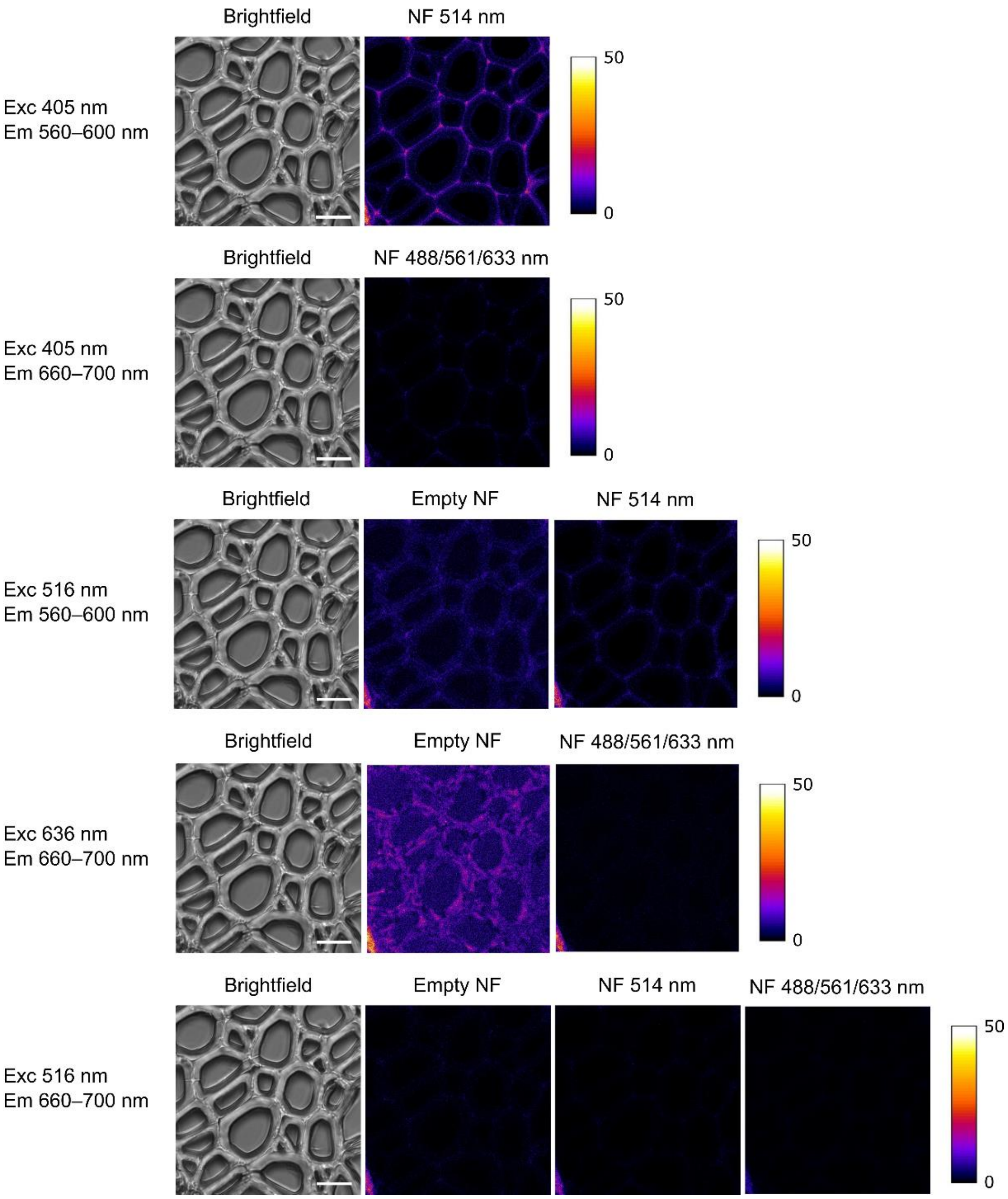

Figure S6. Fluorescence crosstalk between lignin autofluorescence and DyLight Dyes D550 and D633 and effect of applied Notch Filters NF514 and NF488/561/633 nm. Images were not deconvolved. Scale bars correspond to 10 μm.

CBHII binding to α-cellulose (z-stack 7 µm)

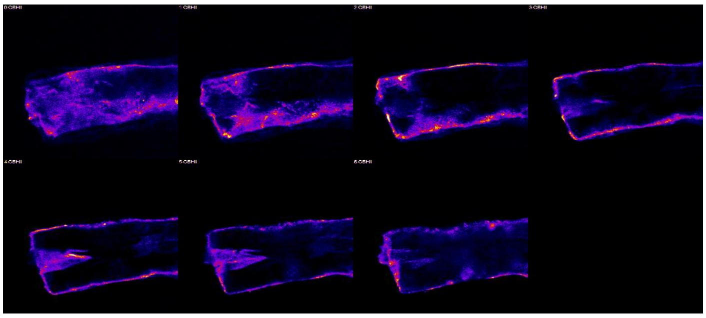

Cel45A binding to α-cellulose (z-stack 7 µm)

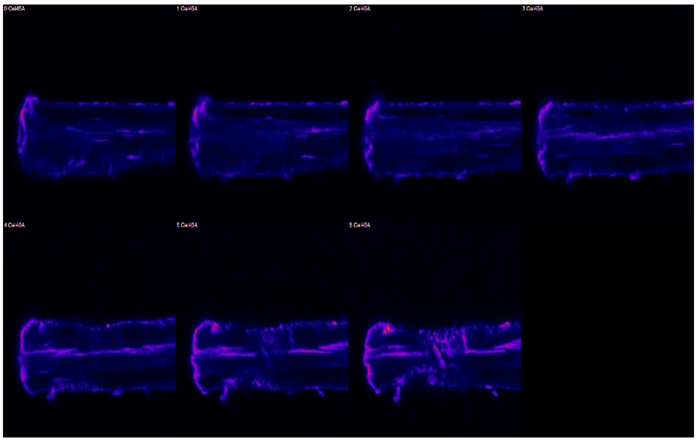

LPMO9D binding to α-cellulose (z-stack 9 µm)

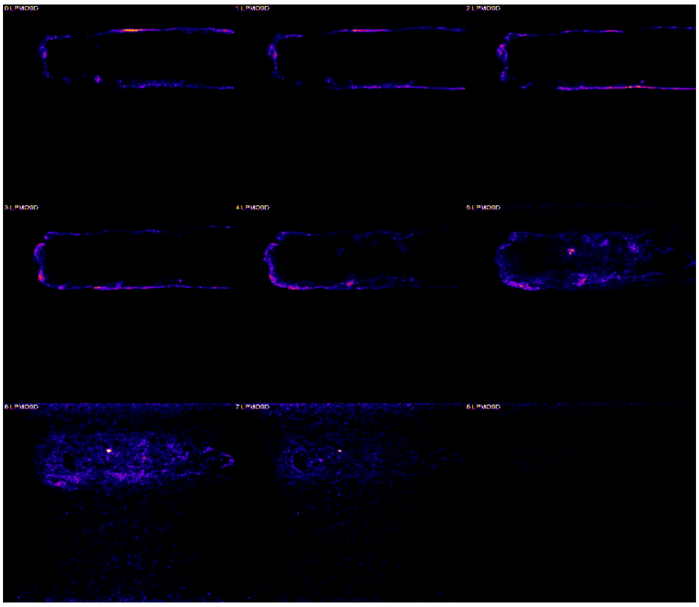

CDH binding to α-cellulose (z-stack 5 μm)

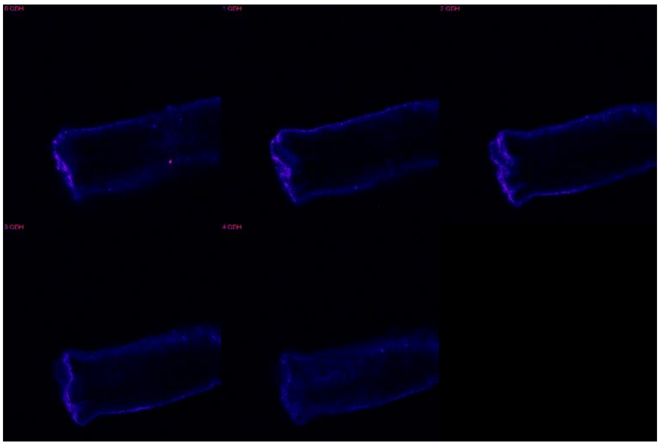

GLOX binding to α-cellulose (z-stack 8 μm)

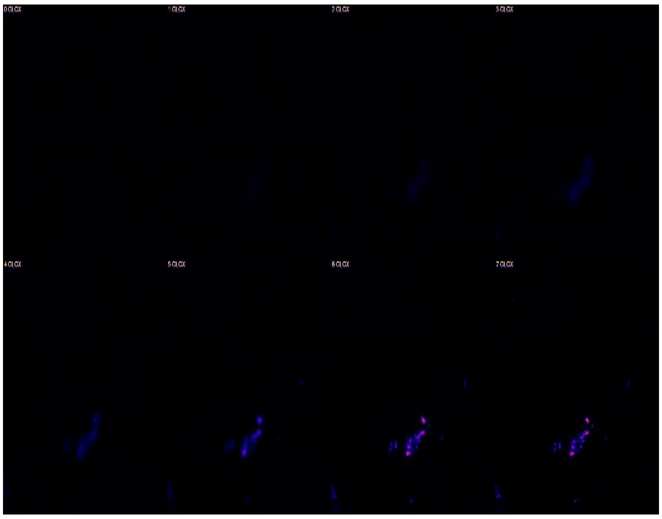

Figure S7. Representative confocal z-stack images of labeled enzymes on α-cellulose. The z-step size was 1 μm and the images are intensity-coded using Fire LUT. Images are displayed with the same fluorescence intensity scale (0–255).

AbCBHII binding to α-cellulose (z-stack 7 μm)

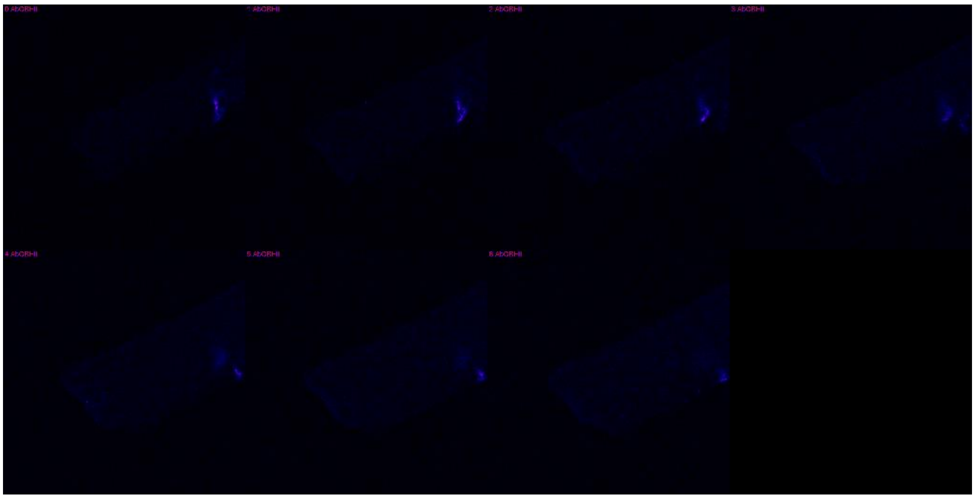

AbCel45A binding to α-cellulose (z-stack 7 μm)

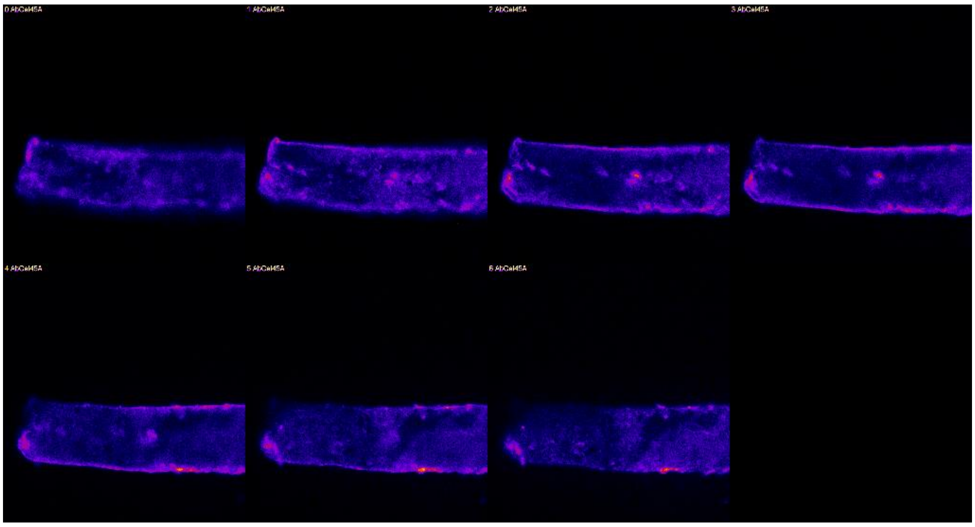

AbLPMO9D binding to α-cellulose (z-stack 6 μm)

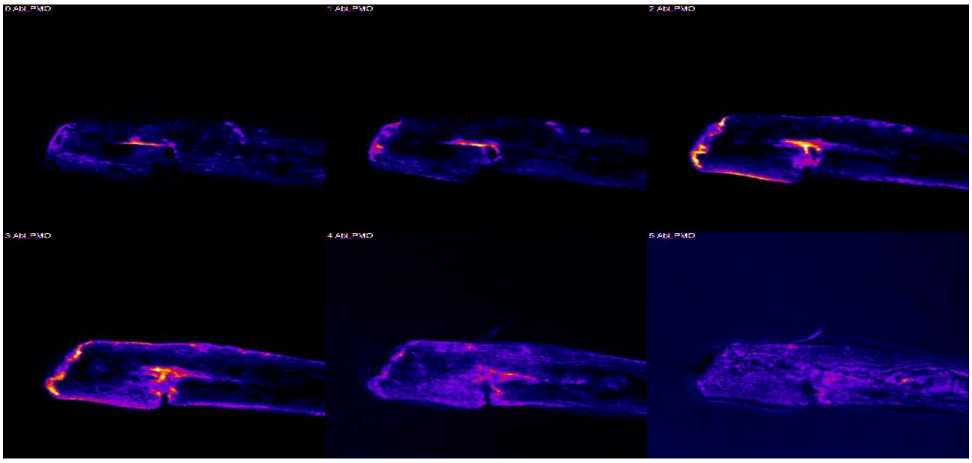

AbCDH binding to binding to α-cellulose (z-stack 6 µm)

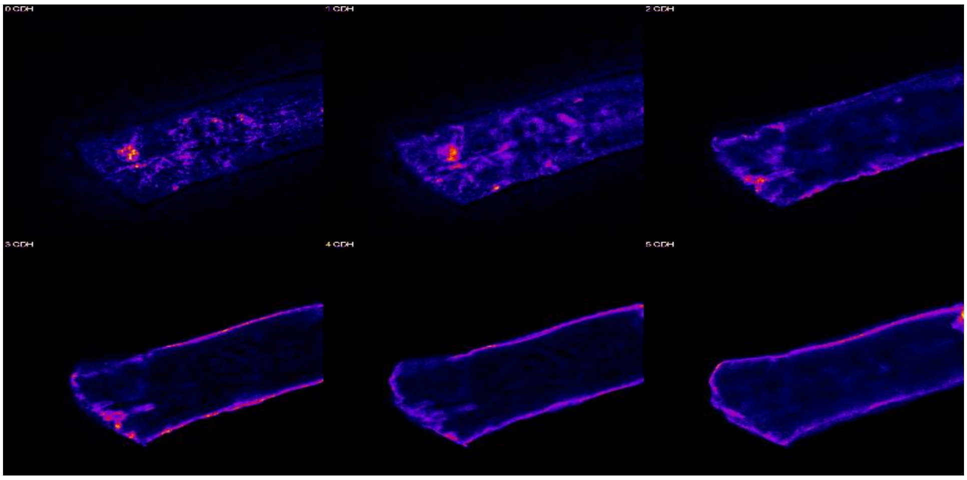

AbGLOX binding to binding to α-cellulose (z-stack 7 µm)

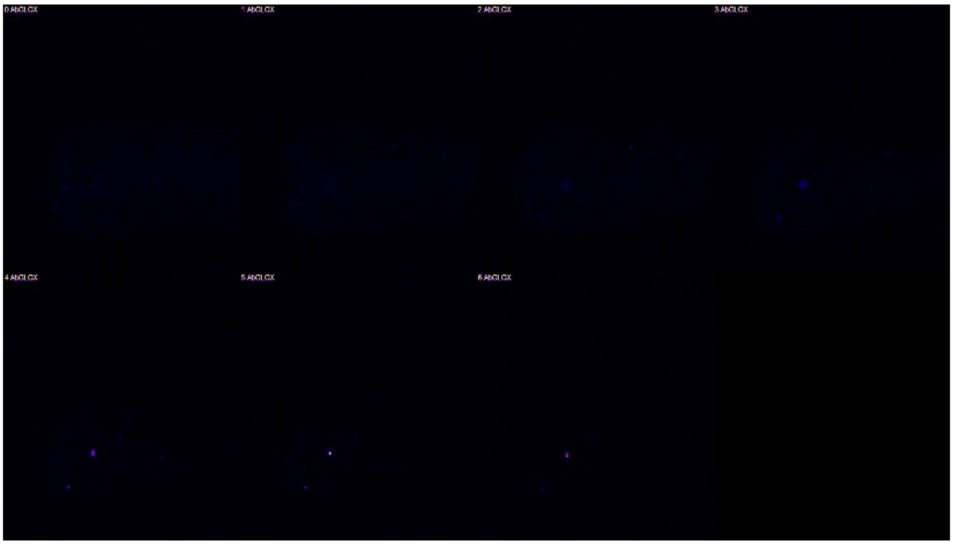

Figure S8. Representative confocal z-stack images of labeled polyclonal antibodies on α-cellulose. The z-step size was 1 µm and the images are intensity-coded using Fire LUT. Images are displayed with the same fluorescence intensity scale (0–255) except for AbCBHII (0–130).

References

1. Igarashi, K.; Uchihashi, T.; Koivula, A.; Wada, M.; Kimura, S.; Penttilä, M.; Ando, T.; Samejima, M. Visualization of Cellobiohydrolase I from *Trichoderma reesei* moving on crystalline cellulose using high-speed atomic force microscopy. *Methods Enzymol.* **2012**, *510*, 169–182, doi:10.1016/b978-0-12-415931-0.00009-4.
2. Igarashi, K.; Ishida, T.; Hori, C.; Samejima, M. Characterization of an endoglucanase belonging to a new subfamily of glycoside hydrolase family 45 of the basidiomycete *Phanerochaete chrysosporium*. *Appl. Environ. Microbiol.* **2008**, *74*, 5628, doi:10.1128/aem.00812-08.
3. Amengual, N.G.; Csarman, F.; Wohlschlager, L.; Ludwig, R. Expression and characterization of a family 45 glycosyl hydrolase from *Fomitopsis pinicola* and comparison to *Phanerochaete chrysosporium* Cel45A. *Enzyme Microb. Technol.* **2022**, *156*, 110000, doi:10.1016/j.enzmictec.2022.110000.
4. Westereng, B.; Ishida, T.; Vaaje-Kolstad, G.; Wu, M.; Eijsink, V.G.H.; Igarashi, K.; Samejima, M.; Ståhlberg, J.; Horn, S.J.; Sandgren, M. The putative endoglucanase PcGH61D from *Phanerochaete chrysosporium* is a metal-dependent oxidative enzyme that cleaves cellulose. *PLoS One* **2011**, *6*, e27807, doi:10.1371/journal.pone.0027807.
5. Wohlschlager, L.; Csarman, F.; Chang, H.; Fitz, E.; Seiboth, B.; Ludwig, R. Heterologous Expression of *Phanerochaete chrysosporiu*m cellobiose dehydrogenase in *Trichoderma reesei*. *Microb. Cell Factories* **2021**, *20*, 1–12, doi:10.1186/S12934-020-01492-0.
6. Wohlschlager, L.; Csarman, F.; Zrilić, M.; Seiboth, B.; Ludwig, R. Comparative characterization of glyoxal oxidase from *Phanerochaete chrysosporium* expressed at high levels in *Pichia pastoris* and *Trichoderma reesei*. *Enzyme Microb. Technol*. **2021**, 145, 109748, doi:10.1016/j.enzmictec.2021.109748.
7. Bao, W.; Usha, S.N.; Renganathan, V. Purification and characterization of cellobiose dehydrogenase, a novel extracellular hemoflavoenzyme from the white-rot fungus *Phanerochaete chrysosporium*. *Arch. Biochem. Biophys.* **1993**, *300*, 705–713, doi:10.1006/abbi.1993.1098.
8. Baminger, U.; Subramaniam, S.S.; Renganathan, V.; Haltrich, D. Purification and characterization of cellobiose dehydrogenase from the plant pathogen *Sclerotium (Athelia) Rolfsii*. *Appl. Environ. Microbiol.* **2001**, *67*, 1766–1774, doi:10.1128/aem.67.4.1766-1774.2001.
9. Daou, M.; Piumi, F.; Cullen, D.; Record, E.; Faulds, C.B. Heterologous production and characterization of two glyoxal oxidases from *Pycnoporus Cinnabarinus*. *Appl. Environ. Microbiol.* **2016**, *82*, 4867, doi:10.1128/aem.00304-16.
10. Martin Hallberg, B.; Henriksson, G.; Pettersson, G.; Divne, C. crystal structure of the flavoprotein domain of the extracellular flavocytochrome cellobiose dehydrogenase. *J. Mol. Biol.* **2002**, *315*, 421–434, doi:10.1006/jmbi.2001.5246.
